# Comparative genomics of *Campylobacter concisus* isolates reveals genetic diversity and provides insights into disease association

**DOI:** 10.1186/1471-2164-14-585

**Published:** 2013-08-28

**Authors:** Nandan P Deshpande, Nadeem O Kaakoush, Marc R Wilkins, Hazel M Mitchell

**Affiliations:** 1 Systems Biology Initiative, School of Biotechnology and Biomolecular Sciences, The University of New South Wales, Sydney 2052, NSW, Australia; 2 School of Biotechnology and Biomolecular Sciences, The University of New South Wales, Sydney 2052, NSW, Australia; 3 Ramaciotti Centre for Gene Function Analysis, The University of New South Wales, Sydney 2052, NSW, Australia

**Keywords:** *Campylobacter concisus*, Comparative genomics, Pathogenesis, Phylogeny, Peptidoglycan, Respiration, *Campylobacter jejuni*

## Abstract

**Background:**

In spite of its association with gastroenteritis and inflammatory bowel diseases, the isolation of *Campylobacter concisus* from both diseased and healthy individuals has led to controversy regarding its role as an intestinal pathogen. One proposed reason for this is the presence of high genetic diversity among the genomes of *C. concisus* strains.

**Results:**

In this study the genomes of six *C. concisus* strains were sequenced, assembled and annotated including two strains isolated from Crohn’s disease patients (UNSW2 and UNSW3), three from gastroenteritis patients (UNSW1, UNSWCS and ATCC 51562) and one from a healthy individual (ATCC 51561). The genomes of *C. concisus* BAA-1457 and UNSWCD, available from NCBI, were included in subsequent comparative genomic analyses. The Pan and Core genomes for the sequenced *C. concisus* strains consisted of 3254 and 1556 protein coding genes, respectively.

**Conclusion:**

Genes were identified with specific conservation in *C. concisus* strains grouped by phenotypes such as invasiveness, adherence, motility and diseased states. Phylogenetic trees based on ribosomal RNA sequences and concatenated host-related pathways for the eight *C. concisus* strains were generated using the neighbor-joining method, of which the 16S rRNA gene and peptidoglycan biosynthesis grouped the *C. concisus* strains according to their pathogenic phenotypes. Furthermore, 25 non-synonymous amino acid changes with 14 affecting functional domains, were identified within proteins of conserved host-related pathways, which had possible associations with the pathogenic potential of *C. concisus* strains. Finally, the genomes of the eight *C. concisus* strains were compared to the nine available genomes of the well-established pathogen *Campylobacter jejuni*, which identified several important differences in the respiration pathways of these two species. Our findings indicate that *C. concisus* strains are genetically diverse, and suggest the genomes of this bacterium contain respiration pathways and modifications in the peptidoglycan layer that may play an important role in its virulence.

## Background

*Campylobacter concisus* has received increasing attention over the last decade and has been described, in a number of publications, as an emergent pathogen of the human intestinal tract [1,2]. *C. concisus* has been isolated from faecal samples of diarrhoeic patients, in some cases contributing to a significant percentage of *Campylobacter* species cultured [3-5]. Moreover, Hess *et al.* have reported a case study of gastroenteritis caused by *C. concisus*[6]. More recently, Nielsen *et al.* reported a high incidence of *C. concisus*, almost as high as that of *C. jejuni/C. coli*, in patients with gastroenteritis from a mixed urban and rural community in Denmark [7]. In a follow-up study [8], the authors found that 80% of *C. concisus* patients and only 32% of *C. jejuni/C. coli* patients had diarrhoea for >2 weeks. Significantly, 6 months following diagnosis, 12% of patients infected with *C. concisus* were diagnosed with microscopic colitis. In contrast, no patient previously diagnosed with *C. jejuni/C. coli* had microscopic colitis. This is of particular significance as previous studies from our group identified, for the first time, a possible association between *C. concisus* and newly diagnosed Crohn’s disease (CD) [9]. Based on a *C. concisus*-specific PCR, a significantly higher prevalence of *C. concisus* DNA was shown to be present in both biopsy and faecal samples of children with newly diagnosed CD than in controls [9,10]. In a further study, we identified 31 *C. concisus* proteins to be immunoreactive in children with CD [11]. Interestingly, a study by Mukhopadhya *et al.* reported the prevalence of *C. concisus* DNA in biopsy specimens from adults with UC to be significantly increased (33.3%; 23/69) as compared with controls (10.8%; 7/65), suggesting that *C. concisus* may also be associated with UC [12].

Investigation of the pathogenic potential of *C. concisus* strains has shown that the bacterium can adhere to human intestinal epithelial cells, however, only some can invade into the cells through transcellular and paracellular mechanisms [13,14]. The transcellular invasion of *C. concisus* strains isolated from chronic intestinal diseases was more than 500-fold higher than that of the other *C. concisus* strains [13,14]. Moreover, host cells infected with *C. concisus* were found to produce high amounts of IL-12, however, only *C. concisus* strains capable of internalising into host cells induced a significantly increased quantity of IFN-γ with respect to controls [13]. These findings, coupled with the regulation of the proteasome, ubiquitination pathways, the Akt signalling pathway and NF-κB inhibitors, pointed towards the activation of the NF-κB pathway by invasive *C. concisus* strains [13]. Further investigation of the difference in invasive potential between strains identified a plasmid containing several virulence determinants, including exotoxin 9 [13,15], which was present in the highly invasive strains but absent in the other strains.

Although the above studies support the role of *C. concisus* as an intestinal pathogen, the isolation of *C. concisus* from healthy individuals, and the failure of some studies to show a significant difference in the prevalence of *C. concisus* in subjects with diarrhoea and healthy controls [1], has raised contention as to the role of *C. concisus* in intestinal disease. While these latter findings would to some degree argue against the role of *C. concisus* in gastroenteritis, the fact that great sequence diversity exists within *C. concisus* strains [3,16] raises the possibility that differences may exist in their pathogenic potential. To further examine the importance of *C. concisus* heterogeneity with respect to disease potential, we sequenced the genomes of six new *C. concisus* strains and performed comparative analyses of these and two known strains, which allowed us to compare strains isolated from three CD, one chronic gastroenteritis, three acute gastroenteritis patients as well as one from a healthy control.

## Results and discussion

### Draft genome assemblies and plasmids of six *Campylobacter concisus* strains

**Figure 1:**
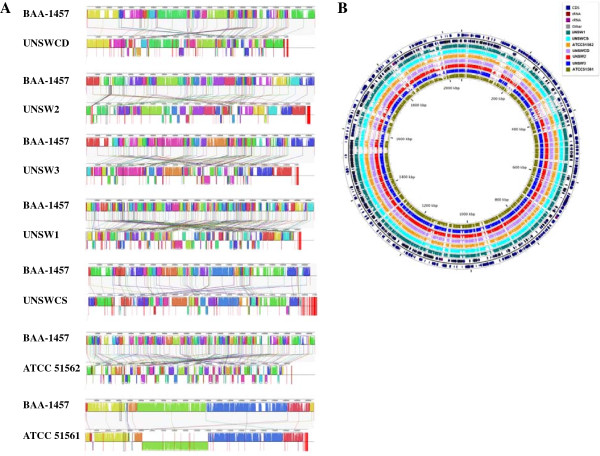
**Synteny based similarities and differences across *Campylobacter concisus *strains. A**: Pairwise comparisons between the reference BAA-1457 genome from NCBI and the *C. concisus* strains sequenced by our group revealed similarities and differences in syntenical placement of genomic blocks. The Mauve alignment tool was used to show paired synteny alignments, which indicate some genome shuffling of *C. concisus* strains. Inversions are indicated by syntenic blocks placed bellow the main axis. **B**: Circular layout of seven assembled genomes against the BAA-1457 reference. The CGView tool used with BAA-1457 as the reference (outer 2 rings) highlights that the reference strain contains regions with high diversity (marked by prominent gaps) when compared to the other seven *C. concisus* strains.

Genomic read-data for the six *C. concisus* strains was generated using a multiplexing approach in a single lane on an Illumina HiSeq sequencing platform, and *de novo* assemblies with varying contig numbers ranging from 28–207 (9–53 scaffolds) were obtained. Two previously sequenced *C. concisus* strains BAA-1457 and UNSWCD, the latter sequenced by our group [15] were also included in our analyses as shown in Table 1. The individual genome sizes varied from 1.81 Mb to 2.11 Mb across the *C. concisus* strains. The Velvet assembly tool was found to produce more compact assemblies with lower contig numbers and higher N50 values (Table 1). Strain-specific sequencing problems were observed for ATCC 51561 and UNSW1 that resulted in their re-sequencing, and thus, the number of reads generated for these two strains vary from the other strains (Table 1). As genomes may undergo large-scale changes during evolution, global alignments using the Mauve alignment tool [17] were performed by pairwise comparison of the seven *C. concisus* strains with the reference BAA-1457 strain (Figure 1A). This analysis revealed varying degrees of genome shuffling (shown by line connections of locally collinear blocks (LCB) across genome pairs) and inversions marked below the reference axis. A high level of similarity and contiguity between the BAA-1457 and ATCC 51561 strains was observed (Figure 1A). A comparative genomic view of the eight *C. concisus* strains was generated using the CGView tool (Figure 1B), which also revealed significant diversity among the strains. Specifically, prominent gaps in gene content were observed in the seven other strains when compared to BAA-1457 (Figure 1B), which is in line with our previous findings [15,18].

**Table 1:** ***Campylobacter concisus *strains used in this study**

**Strain**	**UNSW2**	**UNSW3**	**UNSWCD**	**UNSW1**	**UNSWCS**	**ATCC 51562**	**BAA-1457**	**ATCC 51561**
**Disease**	Crohn’s disease	Crohn’s disease	Crohn’s disease	Gastroenteritis	Gastroenteritis	Gastroenteritis	Gastroenteritis	Healthy
**Disease type**	Chronic	Chronic	Chronic	Chronic	Acute	Acute	Acute	-
**Invasion**	High	High	High	High	Low	Low	No	No
**Adherence**	High	High	High	High	High	Low	High	Low
**Motility**	High	High	High	High	High	Low	Low	Low
**Reads × 2**	17,704,213	18,593,137	N/A	57,904,810	19,805,749	17,541,124	N/A	61,684,406
**Contigs**	114	62	96	77	207	28	1	73
**N50**	89,312	92,608	64,047	117,975	68,143	361,423	2,052,007	111,029
**Scaffolds**	25	25	-	22	53	9	1	23
**Scaffold N50**	184037	229,705	-	195,982	124,567	405,448	2,052,007	176120
**Genome size (Mbp)**	2.01	1.91	1.81	1.94	2.11	1.84	2.05	1.99
**NCBI accession**	ANNJ00000000	ANNE00000000	AENQ00000000	ANNF00000000	ANNG00000000	ANNI00000000	CP000792.1	ANNH00000000
							CP000794.1	
							CP000793.1	

Draft genomes were assembled for six *C. concisus* strains that included two isolated from Crohn’s disease patients (UNSW2 and UNSW3), three from gastroenteritis patients (UNSW1, UNSWCS and ATCC 51562) and one from a healthy individual (ATCC 51561). Genome sequences for UNSWCD (Crohn’s disease) and BAA-1457 (acute gastroenteritis) were retrieved from NCBI and used in the analysis. Relative invasion levels ≥ 0.1% were considered high and <0.01% were considered low. Relative adherence levels ≥ 2% were considered high and <0.5% were considered low. Motility levels ≥ 1.6 cm were considered high and <1.6 cm were considered low.

The 26 genes present in the UNSWCD plasmid [13] were checked for conservation and positioning across the seven other strains in this study. The number of genes found to be conserved varied in each strain, from two genes present in BAA-1457 to 24 genes present in UNSW3 (Table 2). Further analysis revealed that a significant number, sometimes all, of these conserved genes were positioned on the same scaffold and with a high level of synteny for each of the strains (Additional file 1: Table S1), suggesting that in some strains these genes may also be on plasmids. A similar analysis was performed for the genes present on the two plasmids within BAA-1457 (pCCON31, n = 33 and pCCON16, n = 23). For the pCCON31 plasmid, a lower level of conservation and synteny was observed in the other seven strains when compared to the UNSWCD plasmid with the exception of UNSWCS, which had 24/33 genes conserved with 12 of them present on one scaffold (Additional file 1: Table S1). Conservation of genes on pCCON16 was similar among UNSWCD, UNSW1, UNSW2 and UNSW3 (6 or 7 genes) and between ATCC 51562 and UNSWCS (3 or 4 genes), with the same genes being conserved across the strains. ATCC 51561 showed the highest level of conservation and synteny with pCCON16, with 17/23 genes conserved with most being found on 2 scaffolds (Additional file 1: Table S1). Interestingly, the level of pCCON16 conservation within the strains correlated with their level of invasiveness within the host (Table 1), suggesting similar evolutionary trends among strains with similar pathogenic potential.

**Table 2:** **Conservation of genes from UNSWCD and BAA-1457 plasmids across the *Campylobacter concisus *strains**

**UNSWCD plasmid**			**BAA-1457 plasmids**					
**Total genes = 26**			**pCCON31 Total genes = 33**			**pCCON16 Total genes = 23**		
***C. concisus *strain**	**Orthologs**	**No-hits**	***C. concisus *strain**	**Orthologs**	**No-hits**	***C. concisus *strain**	**Orthologs**	**No-hits**
**UNSW2**	9	17	**UNSW2**	8	25	**UNSW2**	6	17
**UNSW3**	24	2	**UNSW3**	1	32	**UNSW3**	7	16
**UNSW1**	22	4	**UNSW1**	4	29	**UNSW1**	6	17
**UNSWCS**	5	21	**UNSWCS**	24	9	**UNSWCS**	4	19
**ATCC 51562**	7	19	**ATCC 51562**	2	31	**ATCC 51562**	3	20
**ATCC 51561**	16	10	**ATCC 51561**	4	29	**ATCC 51561**	17	6
**BAA-1457**	2	24	**UNSWCD**	0	33	**UNSWCD**	7	16

### The pan and core genomes of *Campylobacter concisus*

The pan genome is the supra-genome defining the entire complement of genes within a species, while the core genome is defined as a set of genes found in all sequenced genomes of a species. In addition to the genes from the two strains available in NCBI, the gene sets defined by the RAST server for the six *C. concisus* strains and refined by manual curation were used for defining the pan and core genomes for *C. concisus*. The pan genome was found to consist of 3254 genes with many genes specific for only a sub-set of the strains but dispensable in the other strains. The *C. concisus* core genome consisted of 1556 genes, with a fraction of the *C*. *concisus* core 70/1556 (4.5%) genes encoding hypothetical proteins. Fifty-three KEGG pathways were found to have similar conservation patterns across the eight *C. concisus* strains (Additional file 1: Table S2). Interestingly, none of the 1556 genes from the core were found to be specific to *C. concisus* when analysed against all other bacterial genomes in NCBI.

**Figure 2:**
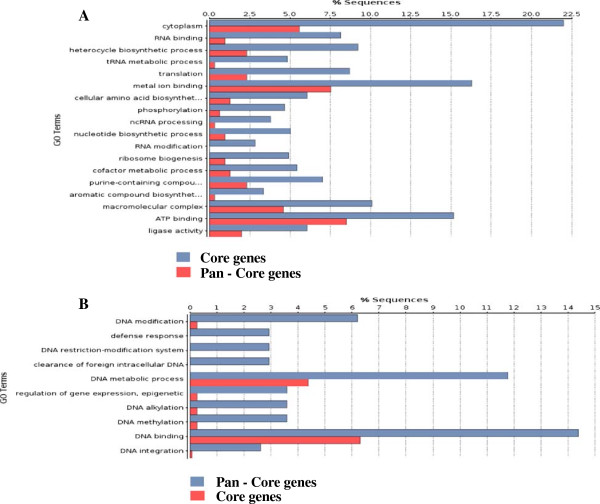
**Enrichment of Gene Ontologies for (A) CORE and (B) PAN genes across *Campylobacter concisus *genomes.**

Comparison of the pan and core genomes identified several gene ontologies to be enriched within each gene set (Figure 2). One indication of the validity of the analysis was the enrichment of RNA processes within the core genome of *C. concisus*, which would be expected given that these processes are conserved across all bacterial genomes. The core genome contained a relatively large number of cytosolic proteins, most likely due to the fact that the cytoplasm encompasses the conserved metabolic machinery of bacteria (tRNA metabolic process is also enriched). This is supported by the fact that ‘metal-ion binding’ is enriched in the core (Figure 2A), as many metabolic enzymes have metal-binding catalytic centres.

Interestingly, in the pan genome, clearance of foreign intracellular DNA and other DNA-related processes such as DNA integration and DNA restriction-modification were enriched (Figure 2B), suggesting that differences exist in the efficiency of these *C. concisus* strains to survive phage attacks. This is supported by the enrichment of ‘defence response’ within the pan genome, and would indicate that this process is not conserved among all *C. concisus* strains.

### Phylogenetic analysis based on ribosomal RNA genes

**Figure 3:**
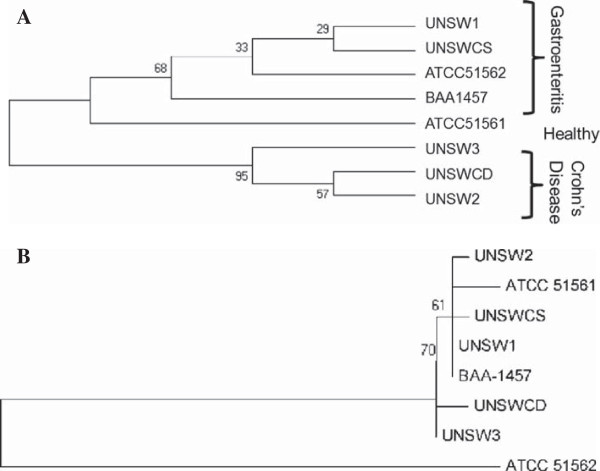
**Phylogenetic analysis of (A) 16S and (B) 23S ribosomal RNA genes for the eight *Campylobacter concisus *strains.**

Phylogenetic trees were generated for the 16S rRNA and 23S rRNA genes of the eight *C. concisus* strains to examine their evolutionary relationships. Interestingly, the tree based on the 16S rRNA gene sequence grouped the strains isolated from patients with CD together (Figure 3A), suggesting that this gene may be used as a marker of genetic heterogeneity within this species. In contrast, phylogeny based on the 23S rRNA gene sequence showed no categorisation based on any of the phenotypes (Figure 3B). The addition of 20 other *Campylobacter* sequences from a range of *Campylobacter* species into each of the trees did not change the grouping of the strains for both trees (data not shown). These results suggest that while there may be some evolutionary role at play in the involvement of *C. concisus* strains in particular diseases, the most likely factors contributing to differences in pathogenic potential are specific genes acquired by the strains through mechanisms such as horizontal gene transfer. Moreover, these results support the findings that phylogenies based on the 16S and 23S rRNA genes do not always group bacteria in a similar manner [19].

### Inter-strain differences and phenotype-based analyses

#### Gene and metabolic differences within the strains

In order to determine the level of inter-strain diversity within *C. concisus*, a comparison of the eight strains was performed to identify genes specific to each strain and to strain pairs (Additional file 1: Table S3 and Table S4). Significant differences in the number of specific genes per strain were observed, and many of these genes were found to be syntenic (Additional file 1: Table S3 and Table S4), thus, indicating the possible acquisition of these strain-specific genes by horizontal gene transfer. Interestingly, the relative percentage of genes unique to each strain was found to group the isolates into three categories, low (<1%), medium (1.2 to 2.0%) and high (>2.5%), and this classification corresponded to non-pathogenic, chronic and acute strains, respectively (Additional file 1: Table S5). These findings suggest an optimal level of acquired genes for the pathogenic efficiency of *C. concisus*, whereby the strain from the healthy subject had a lower number of strain-specific genes relative to its genome size while the strains from acute disease had a higher number when compared to the strains with high pathogenic potential (chronic disease).

Several differences were identified within the metabolic machinery of these eight strains (Additional file 1: Table S6). For example, four of the eight strains contained two genes involved in tetrathionate respiration, a form of respiration which has been shown to give *Salmonella* strains a competitive advantage in the intestine [20]. Interestingly, the genome of ATCC 51561, isolated from the healthy control, was the only strain to contain an aerotaxis receptor which may increase its ability to monitor oxygen concentrations. Moreover, the genomes of both ATCC 51561 and ATCC 51562 contained the phosphate transport system pstABCS, which has been related to the ability of *Shigella* strains to translocate from cell to cell [21]. This system may have been acquired by these strains from their interaction with *Shigella* species within the oral cavity or intestine. The genomes of several strains contained elements of type IV and VI secretion systems, however, these systems appear to be incomplete which suggests that these systems may not be of importance to *C. concisus*.

In addition, a single gene encoding a DUF2920 superfamily bacterial protein with unknown function (also annotated as a motility accessory factor and a carbonic anhydrase in other *Campylobacter* species) was found to be well conserved across all strains isolated from patients with intestinal disease, but was absent in the strain from the healthy subject, making it an interesting target for future examination.

#### Genes associated with adherence potential

Searches for genes present in the strains with high adherence and absent in those with low adherence identified the sodium-hydrogen antiporter NhaC. Sodium-hydrogen antiporters have been linked to regulation of sodium concentrations and pH balance within cells by using H^+^[22]. These proteins convert the proton motive force to a sodium motive force for efflux of Na^+^ ions. It is well-known that the flagella of *Campylobacter* species, including *C. concisus*[13,14], play a major role in the adherence to host cells, and bacterial flagella are driven by a proton motive force [23]. Thus, it is conceivable that the absence of NhaC from some *C. concisus* strains may influence the proton motive force, and thus, influence the strength of flagellar adherence to host microvilli. While this may suggest the involvement of NhaC in the more highly adherent phenotype, other antiporters such as NhaA were identified in the strains with low adherence. Moreover, the fact that strain BAA-1457 has high adherence and low motility would indicate that other factors are involved in this phenotype. Further work is required to establish an association between sodium-hydrogen antiporters and bacterial adherence to host cells.

#### Genes associated with invasive potential

**Figure 4:**
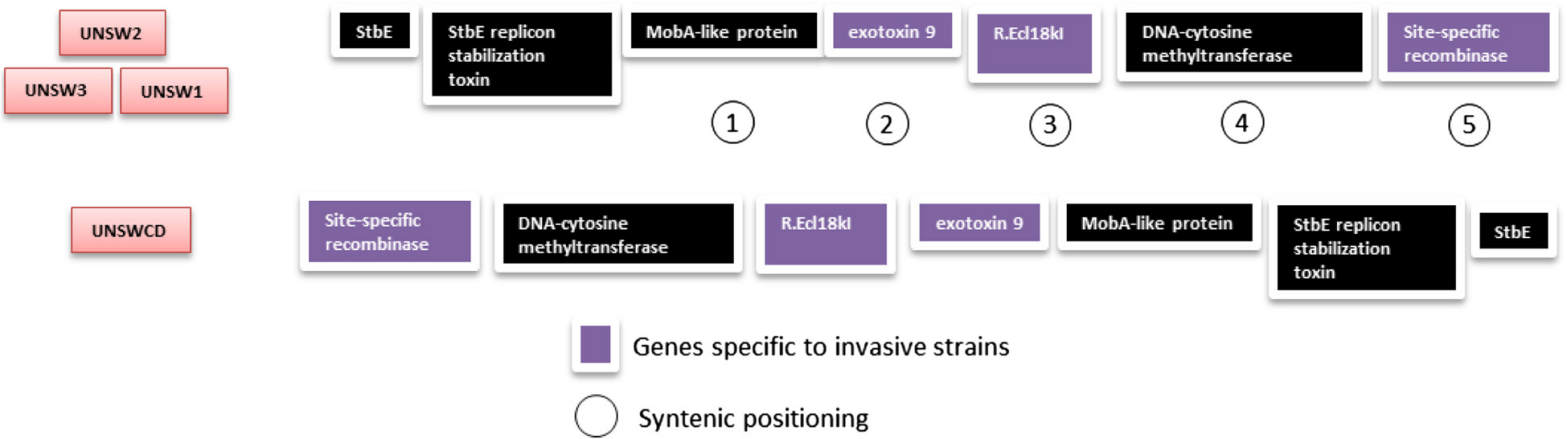
**Syntenic placement of specific conserved plasmid genes in the genomes of invasive strains.** All genes were placed on a single scaffold in each species.

Preliminary investigations by our group to identify possible factors responsible for the increased invasive potential of some *C. concisus* strains revealed the presence of a plasmid with conserved elements only in the highly invasive strains [13]. In this study, we extended this analysis by comparing the complete sequence data from all 8 strains. In agreement with our previous study, the exotoxin 9 gene was identified only in the highly invasive strains. However, BLAST searches across all the strains determined that several genes located on this plasmid were present in strains with low or no invasion potential. The syntenic conservation of the seven genes within the UNSWCD plasmid was found only in the highly invasive strains (Figure 4), however only three of these genes (exotoxin 9, site-specific recombinase and a restriction endonuclease) were specific to these strains (Additional file 1: Table S1). The DNA-cytosine methyltransferase which lies between the restriction endonuclease and the site-specific recombinase was also found in strains with low invasive potential (UNSWCS and ATCC 51562), however, not in non-invasive strains (BAA-1457 and ATCC 51561) (Figure 4, Additional file 1: Table S1).

The function of the proteins encoded by these plasmid genes has the potential to provide insights into their conservation patterns. For example, the presence of the MobA-like protein and the StbD/E toxin antitoxin system in the other strains most likely relates to their global role in ensuring that daughter cells inherit DNA properly [24]. In contrast, further BLAST searches revealed the exotoxin 9 to be the possible helicase DnaI, and its conserved synteny with the restriction endonuclease, recombinase and DNA methyltransferase suggests a combined function for these proteins within the organism, more likely associated with the ability to survive within host cells than its entry into host cells. Of interest, the sequence of the exotoxin 9 had very high homology (88-92%) to a replicative DNA helicase from *Lachnoanaerobaculum saburreum* and related species. These organisms have been isolated from the oral cavity and intestinal biopsies of humans, and produce both H_2_S and NH_3_[25], which are properties shared with *C. concisus* (discussed below).

Another single gene on the chromosomal DNA annotated as a hypothetical protein was also found to be specific to the highly invasive *C. concisus* strains. Following BLAST searches, this protein appears to be a subunit of the molybdopterin synthase enzyme, which synthesises molybdenum cofactor (MoCo). This enzyme is important for respiratory nitrate reductase activity and sulfur metabolism, suggesting that the highly invasive strains may have a competitive advantage over other strains through other forms of respiration.

### Phylogeny and SNP analysis of host-related pathways

**Figure 5:**
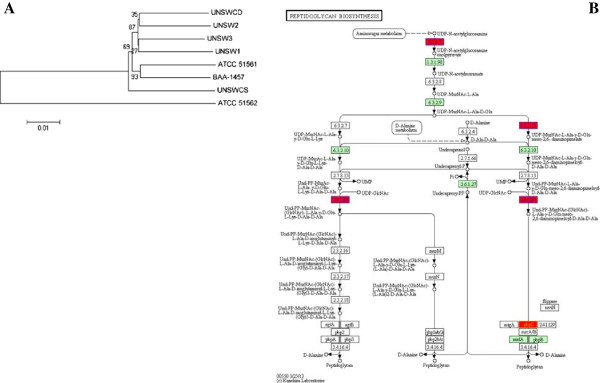
**Analyses of the peptidoglycan biosynthesis pathway within the eight *C. concisus *strains. A**: Phylogenetic analysis of the concatenated sequences of the peptidoglycan biosynthesis pathway within the eight *C. concisus* strains. **B**: Genes in the peptidoglycan biosynthesis pathway with SNPs are highlighted in red.

Following the identification of specific genes associated with *C. concisus* phenotypes, phylogenetic analyses were performed on important host-related pathways that were conserved within the eight strains. This was conducted in order to establish whether specific changes within the genes, rather than the presence or absence of the genes was an important factor in the modulation of the pathogenic potential of *C. concisus*. The pathways analysed were bile efflux, flagellar biosynthesis, chemotaxis, lipopolysaccharide biosynthesis, peptidoglycan biosynthesis, and the sulfur relay system, as these pathways have been previously associated with the pathogenic potential and fitness of bacteria. The majority of these pathways did not group the strains based on any known properties or phenotypes (Additional file 1: Figure S1) except for peptidoglycan biosynthesis which clustered the highly invasive strains apart from the rest of the strains (Figure 5A). This suggests that the peptidoglycan layer of these strains may be different, and may influence their ability to survive within host cells. Stintzi *et al.* have shown that *C. jejuni* extensively remodels its envelope *in vivo* by differentially expressing its membrane proteins and by modifying its peptidoglycan and glycosylation composition [26]. Moreover, Frirdich *et al.* found that mutation of the peptidoglycan DL-carboxypeptidase pgp1 of *C. jejuni* resulted in a loss of spiral morphology, deficiency in chicken colonisation, defects in biofilm formation and motility, enhanced secretion of IL-8 and increased activation of Nod1 [27], providing further evidence of the involvement of the peptidoglycan layer of *Campylobacter* species in their pathogenic potential.

The sequences of the genes involved in the above pathways were screened for non-synonymous SNPs that may be associated with *C. concisus* phenotypes (Table 3). Twenty-one non-synonymous SNPs, of which 10 were present within known functional domains (Table 3), were identified within the bile efflux pathway, peptidoglycan biosynthesis, lipopolysaccharide biosynthesis and the sulfur relay system to be associated with the phenotypes presented in Table 1. Six amino acid changes detected in the outer membrane component of the bile efflux pathway CmeD were associated with the highly invasive phenotype (n = 2), the highly motile phenotype (n = 2) and the highly adherent phenotype (n = 2) (Table 3). In addition, two amino acid changes in the transporter component of this pathway CmeB were associated with motility (n = 1) and adherence (n = 2). Diversity in the genes involved in this bile efflux pathway has been reported in *C. jejuni* and *C. coli* by Cagliero *et al.*, who suggested that these variations may impact on the function of this pathway [28]. Thus, it is possible that specific amino acid changes within the outer membrane and transporter components of this pathway may play a role in the pathogenesis of *C. concisus*. Of particular interest, amino acid changes within the flagellar P-ring protein PbpC, which attaches the flagellum to the peptidoglycan layer, were associated with motility and adherence (Table 3, Figure 5B). Moreover, the two enzymes MurG and MurG transferase involved in peptidoglycan biosynthesis also contained amino acid changes that were associated with the more adherent phenotype of *C. concisus* (Figure 5B), providing further evidence that both the flagellum and peptidoglycan layer of *C. concisus* are important for the pathogenesis of the bacterium.

**Table 3:** **Non-synonymous SNPs across *C. concisus *strains displaying categorisation based on known phenotypes**

**Gene ID**	**aa position**	**bp position**	**UNSW2**	**UNSW3**	**UNSW1**	**UNSWCD**	**UNSWCS**	**ATCC51562**	**ATCC51561**	**BAA1457**	**Phenotype**	**Domains**			
												**Name**	**Type**	**start**	**end**
**Bile efflux (Cme) pathway**															
CmeB	848	2544	GAC:D	GAC:D	GAT:D	GAT:D	GAC:D	GAG:E	GAG:E	GAT:D	**Adherence**	ACR_tran	PFAM	2	1024
CmeB	519	1557	AGG:R	AGG:R	AGG:R	AGG:R	AGG:R	AAG:K	AAG:K	AAG:K	**Motility**	ACR_tran	PFAM	2	1024
CmeD	227	681	AAG:K	AAG:K	AAG:K	AAG:K	AGG:R	AGA:R	AGG:R	AGG:R	**Invasive**	NA			
CmeD	229	687	AGT:S	AGT:S	AGT:S	AGT:S	AAT:N	AAT:N	AAT:N	AAT:N	**Invasive**	NA			
CmeD	233	699	TCT:S	TCT:S	TCT:S	TCT:S	TCT:S	TTT:F	TTT:F	TTT:F	**Motility**	NA			
CmeD	234	702	TTA:L	TTA:L	TTA:L	TTA:L	TTA:L	ATA:I	ATA:I	ATA:I	**Motility**	NA			
CmeD	163	489	CCA:P	CCA:P	CCA:P	CCA:P	CCA:P	GCG:A	GCA:A	CCA:P	**Adherence**	OEP	PFAM	32	201
CmeD	362	1086	AGA:R	AGA:R	AGA:R	AGA:R	AGA:R	AAA:K	AAA:K	AGA:R	**Adherence**	NA			
**00540|Lipopolysaccharide biosynthesis**															
CCC13826_0577	161	483	AAC:N	AAC:N	AAC:N	AAC:N	AAC:N	AGC:S	AGC:S	AAC:N	**Adherence**	NA			
CCC13826_0577	161	483	AAC:N	AAC:N	AAC:N	AAC:N	AAC:N	AGC:S	AGC:S	AAC:N	**Adherence**	PfkB	PFAM	6	298
CCC13826_0470	104	312	ATT:I	ATT:I	ATT:I	ATT:I	ATT:I	ACT:T	ACT:T	ATT:I	**Adherence**	Glyco_transf_9	PFAM	75	290
**00550|Peptidoglycan biosynthesis**															
CCC13826_1265	202	606	AGA:R	AGA:R	AGA:R	AGA:R	AGA:R	AAG:K	AAA:K	AAA:K	**Motility**	EPSP_synthase	PFAM	8	409
CCC13826_1740	184	552	GTA:V	GTA:V	GTA:V	GTA:V	GTA:V	ATG:M	ATG:M	GTA:V	**Adherence**	NA			
CCC13826_1740	184	552	GTA:V	GTA:V	GTA:V	GTA:V	GTA:V	ATG:M	ATG:M	GTA:V	**Adherence**	Mur_ligase_M	PFAM	64	257
CCC13826_1242	26	78	GGC:G	GGC:G	GGC:G	GGC:G	GGC:G	GAT:D	GAC:D	GGT:G	**Adherence**	Glyco_transf_28	PFAM	2	139
CCC13826_1242	26	78	GGC:G	GGC:G	GGC:G	GGC:G	GGC:G	GAT:D	GAC:D	GGT:G	**Adherence**	NA			
CCC13826_1254	361	1083	CTG:L	CTA:L	CTA:L	CTA:L	CTG:L	ATA:I	ATA:I	ATA:I	**Motility**	NA			
CCC13826_1254	361	1083	CTG:L	CTA:L	CTA:L	CTA:L	CTG:L	ATA:I	ATA:I	ATA:I	**Motility**	Transpeptidase	PFAM	295	532
CCC13826_1254	636	1908	GTC:V	GTT:V	GTT:V	GTC:V	GTT:V	ATT:I	ATT:I	GTT:V	**Adherence**	NA			
CCC13826_1254	636	1908	GTC:V	GTT:V	GTT:V	GTC:V	GTT:V	ATT:I	ATT:I	GTT:V	**Adherence**	NA			
**Sulfur relay system**															
CCC13826_1163	132	396	GAT:D	GAT:D	GAT:D	GAT:D	GAC:D	GAA:E	GAG:E	GAC:D	**Adherence**	Aminotran_5	PFAM	3	366
**N-glycosylation pathway**															
PglB_699	20	60	ATC:I	ATC:I	ATC:I	ATC:I	ATC:I	CTT:L	CTT:L	CTT:L	**Motility**	STT3	PFAM	10	503
PglI	19	57	AAA:K	AAA:K	AAA:K	AAA:K	AAA:K	CAA:Q	CAA:Q	AAA:K	**Adherence**	Glycos_transf_2	PFAM	7	123
PglI	36	108	GTT:V	GTT:V	GTT:V	GTT:V	GTT:V	ATA:I	ATA:I	ATA:I	**Motility**	Glycos_transf_2	PFAM	7	123
PglJ	312	936	GAT:D	GAT:D	GAT:D	GAT:D	GAG:E	GAG:E	GAG:E	GAG:E	**Invasive**	Glycos_transf_1	PFAM	195	351

The presence of the SNPs on functional domains within the protein sequence was also identified.

Analysis of *N*-linked glycans has revealed that *C. concisus,* along with *Campylobacter fetus*, *Campylobacter hyointestinalis*, *Campylobacter lanienae*, *Campylobacter sputorum*, display differing arrangements of Hex and HexNAc sugars to other *Campylobacter* species, and that *C. concisus* contains a residue of 217 Da not found in all other *Campylobacter* species tested [29]. Given this, our phylogeny and SNP analysis was extended to the 13 genes characterised within the *N*-glycosylation pathway and present in the *C. concisus* strains (*pglABCDEFGHIJK*, *wbpO* and *galE*). While the phylogenetic analysis did not group the strains according to a specific phenotype (Additional file 1: Figure S1), a further 4 non-synonymous SNPs were identified, all of which were present within known functional domains (Table 3). As *N*-linked glycosylation of surface proteins appears to enhance *C. jejuni* fitness by protecting bacterial proteins from cleavage by gut proteases [30], differences within this pathway may influence the role *C. concisus* strains play within the gut.

### Comparative genomic analyses of *Campylobacter concisus* and *Campylobacter jejuni*

**Figure 6:**
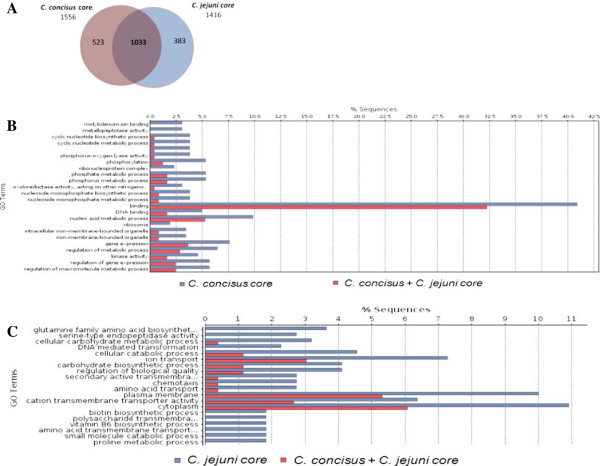
**Comparative genomic analyses of *Campylobacter concisus *and *Campylobacter jejuni*. A**: The core genomes of *C. concisus* and *C. jejuni* were identified. **B**: Enrichment of genes specific to *C. concisus* core genome. **C**: Enrichment of genes specific to *C. jejuni* core genome. Enrichment tests of all Gene Ontology terms were analysed using Fisher’s Exact Test with Multiple Testing Correction of FDR (Benjamini and Hochberg).

An important recent study by Nielsen *et al.* compared the characteristics of *C. jejuni* and *C. concisus* infection within humans [8], and found that while infection with *C. jejuni* was more aggressive, it lasted for a shorter time period than infection with *C. concisus.* Moreover, six months following diagnosis, 12% of patients infected with *C. concisus* were diagnosed with microscopic colitis [8], whereas no *C. jejuni* patients were diagnosed with non-infective colitis. Thus, to identify possible factors involved in the differences between *C. concisus* and *C. jejuni* infections, a comparative analysis of the eight genomes of *C. concisus* with the nine available genomes of *C. jejuni* was performed (Figure 6, Additional file 1: Table S6). While the core genome of the *C. concisus* strains was defined to be 1556 genes, the *C. jejuni* core genome contained 1416 genes. Comparison of the two core genomes identified 1033 genes that were shared between the two species, while 523 genes were specific to the *C. concisus* core and 383 specific to the *C. jejuni* core (Figure 6A). Enrichment analysis of the core genes specific to each species were performed using Blast2Go (Figure 6B, C), and complemented with pathway analyses of all genomes using the KEGG database. Evidence that the genome assembly was correct was the absence of the catalase gene within all *C. concisus* genomes and the presence of this gene within the *C. jejuni* genomes, which is in line with the fact that *C. concisus* is a catalase-negative organism while *C. jejuni* is catalase-positive.

This analysis identified a possible competitive advantage of *C. jejuni* in terms of iron acquisition, as the genomes of this bacterium contained both the ferritin FtnA involved in iron storage, and the Fe^3+^ transport system FbpABC, which were all absent in the *C. concisus* strains. Specificity towards iron was also observed by the presence of the Fe/Mn superoxide dismutase in *C. jejuni*, whereas both the Cu/Zn and Fe/Mn superoxide dismutases were present in *C. concisus*. One significant difference was identified within the peptidoglycan biosynthesis pathway, whereby only *C. concisus* was found to contain the flagellar P-ring protein PbpC. This finding is of particular interest given our previous observation that two SNPs within *pbpC* were associated with motility and adherence (Table 3). Moreover, the fact that the peptidoglycan biosynthesis pathway clusters the highly invasive *C. concisus* strains together when compared to the other *C. concisus* strains (Figure 5), would suggest that differences in this pathway may significantly influence the pathogenic potential of *C. concisus*.

The C4-Dicarboxyrate transport system DctPQM was present within the genomes of all *C. concisus* strains and absent in the *C. jejuni* genomes (Additional file 1: Table S6). This system has been reported to be involved in sensing and differentiating between aerobic and anaerobic respiration [31], which may relate to the fact that *C. concisus* can grow anaerobically whereas *C. jejuni* cannot. This finding is in line with the identification of major differences within the respiration pathways of these two organisms, specifically those related to nitrogen and sulfur respiration. For example, genomes of the *C. concisus* strains all contained the nitric oxide reductase NorB and the nitrous oxide reductase NosZ, which were absent in the genomes of the *C. jejuni* strains. Significantly, *C. jejuni* strains contained the two associated proteins NrfA and NrfH involved in nitrite respiration, whereby NrfH functions to anchor NrfA into the membrane [32]. In contrast, *C. concisus* contains NrfC and NrfD which have been linked to both nitrite and sulfite respiration [33,34]. Moreover, while both organisms contain NapG and NapH, *C. concisus* also contains a NapC/NirT cytochrome *c* family protein, which is absent in *C. jejuni*. In addition to these findings, we found that in *C. jejuni* 2-oxoglutarate is converted to L-glutamate through the glutamate synthase GltD, which is then converted to L-1-Pyrroline-5-carboxylate through the activity of the proline dehydrogenase PutA. In contrast, in *C. concisus* the glutamate dehydrogenase GdhA produces ammonia through its conversion of L-glutamate to 2-oxoglutarate. The hydroxylamine reductase Hcp (present in *C. concisus*; absent in *C. jejuni*) can employ ammonia, water and an acceptor to produce hydroxylamine, which can then be converted to nitrite through the activity of hydroxylamine oxidase Hao (present in *C. concisus*, absent in *C. jejuni*). This nitrite may feed into the nitrite respiration pathway, thereby giving *C. concisus* a means to respire through the amino acid L-glutamate.

In addition to nitrogen-associated respiration, the genomes of the *C. concisus* strains had enzymes within the sulfur metabolism pathway that were absent in *C. jejuni*, most importantly a sulfite reductase which has the ability to convert sulfite to hydrogen sulfide (Additional file 1: Table S6). This is of interest as high levels of H_2_S have been detected in patients with UC [35], and *C. concisus* has been associated with UC [12].

These above differences in the respiration pathways are supported by the finding that molybdenum ion binding is enriched in the *C. concisus* core genome when compared to the *C. jejuni* core genome (Figure 6B), as molybdenum enzymes catalyse the oxidoreduction of certain small molecules, as part of the regulation of nitrogen, sulfur and carbon cycles [36]. Indeed, the importance of these enzymes in the overall physiology of *C. concisus* is highlighted by the fact that the highly invasive strains shared a molybdopterin synthase that was absent in the other strains, and molybdopterin and molybdenum combine to form the molybdenum cofactor.

## Conclusions

This study has confirmed the high genetic diversity observed among *C. concisus* isolates, but more importantly has identified several factors pertaining to the pathogenic potential of the emerging pathogen *C. concisus*. In particular, the peptidoglycan layer of this bacterium and conserved elements within the highly invasive strains such as the exotoxin 9 may play an important role in its virulence. Finally, novel differences within the respiration pathways of the well-known pathogen *C. jejuni* and the emerging pathogen *C. concisus* were identified, which may provide insights into their growth within the environment and the host.

## Methods

### Bacterial strains and DNA preparation

The six *C. concisus* strains sequenced in this study were isolated from patients with CD (UNSW2 and UNSW3), chronic gastroenteritis (UNSW1), acute gastroenteritis (UNSWCS and ATCC 51562) and a healthy subject (ATCC 51561). ATCC 51562 and ATCC 51561 were purchased from the American Type Culture Collection. UNSW1, UNSW2, UNSW3 and UNSWCS were isolated as part of previous study [13] which was approved by the Research Ethics Committees of the University of New South Wales and the South East Sydney Area Health Service-Eastern Section, Sydney (Ethics No.: 06/164). Written consent was obtained from all subjects, or their guardians, participating in that study. Information on the strains is listed in Table 1. *C. concisus* strains were grown on Horse Blood Agar (HBA) plates [Blood Agar Base No. 2 supplemented with 6% defibrinated horse blood (Oxoid)], and incubated at 37°C under microaerobic conditions with H_2_ (generated using *Campylobacter* Gas Generating Kits (Cat. #. BR0056A, Oxoid)) for 48 h. Bacterial DNA was extracted using the Puregene Core Kit (Qiagen) according to the manufacturer’s instructions.

#### Sequencing, assembly and annotation

Genome sequencing of the six *C. concisus* strains was performed using a single multiplexed lane in a Hi-Seq Illumina sequencer. The libraries were prepared using the TruSeq DNA sample preparation kit (Illumina) following the manufacturer’s instructions. Briefly, 1 μg of DNA was sheared using the Covaris, followed by end-repair, A-tailing and ligation of adapters. Size selection was performed with the Pippin prep (Sage Science) selecting for 300–400 bp inserts, which were amplified using 10 cycles of PCR. The libraries were multiplexed in two lanes of the HiSeq 2000 (Illumina) and sequenced as 100 bp paired-end reads. Customised automated python scripts were developed to run two assembly programs, Velvet v1.2.08 and SOAPdenovo v1.05, and the overlap parameter k-mer varied between 51–91 to get an optimised assembly. The scaffolding tool SSPACE-BASIC-2.0_linux-x86_64 [37] was employed by providing the average fragment size of 320 bp with a tolerance of ± 50 bp, and the forward-reverse orientation of the paired-end reads to generate compact scaffolded assemblies for the individual genomes. The Rapid Annotation using Subsystem Technology (RAST) [38], a service for annotating bacterial and archaeal genomes, was used for gene definition and annotation for the individual assemblies. Genome scale alignments were performed using the Mauve alignment tool [17] and CGView [39]. Genomic data for already sequenced *C. concisus* strains BAA-1457 and UNSWCD were obtained from the GenBank repository (Table 1). The UNSWCD strain has previously been sequenced by our group [15].

#### Investigation of plasmids in the assembled genomes

BLAST searches were performed using the genes from the previously identified plasmids within *C. concisus* strains UNSWCD and BAA-1457 to check for conservation in the sequenced *C. concisus* genomes. Detailed annotations and mapping of the gene synteny of the conserved genes on individual *C. concisus* genomes was done using custom scripts. Moreover, a set of all possible genes from plasmids identified in epsilon-Proteobacteria was downloaded from NCBI. The presence of these genes in the six *C. concisus* strains was investigated using BLAST searches.

#### Defining the core and pan genomes

The core genome for the eight *C. concisus* strains was defined using an iterative BLAST method. The UNSWCD proteome from NCBI was used as the starting reference genome. These sets of proteins were put through blast (tblastn, 40% homology and 40% length hit) in a sequential order against the rest of the *C. concisus* genomes. Customised scripts parsed out the intersecting genomes during each of the iterations, and thus, the genes found to be conserved in all the *C. concisus* genomes formed the core genome. The pan genome (that is the set of genes found in at least one *C. concisus* strain) was defined using a similar iterative BLAST strategy. All the genes not in the core were combined and a non-redundant set of genes was defined from the above group by performing an all versus all BLAST search. This gene set was then added to the core gene set to get the final pan genome for *C. concisus*.

#### Enrichment analysis using blast2GO

Functional enrichment analyses of the core and pan genomes were performed using the annotation and analysis tool Blast2GO [40]. Following annotation, the statistical analysis package in Blast2GO which uses Fisher’s exact test with multiple testing correction of false discovery rate [41] was applied and enrichment graphs were generated. A similar method was employed for the analysis of gene ontology enrichment across *C. concisus* and *C. jejuni*.

#### Multi-species, multiple pathway comparisons

A multi-species, multi-pathway comparison utility was developed for filtering KEGG pathways displaying similar as well as varying conservation patterns across sequences bacterial species of interest. The aim was to develop an unbiased method to analyse all KEGG pathways across a given set of species without having any prior knowledge about their possible biological importance in the group of species under study. Similar to KAAS [42] (which gives functional annotation of genes by BLAST comparisons against the manually curated KEGG GENES database), the genes defined for individual *C. concisus* strains were subjected to BLAST alignments against the KEGG GENES database to obtain KO identifiers. The KO identifiers were then mapped onto KEGG pathways. Customised scripts were written to allow comparison for individual KEGG pathways at gene component levels across selected species, to mark their conservation patterns. Pathways with complete or partial conservation for the given set of *C. concisus* and *C. jejuni* strains included in this analysis were separated out for further analysis.

#### Phylogeny and SNP analysis

Phylogenetic trees were generated using the MEGA 5.05 tool [43] by applying the neighbour-joining method with 1000 bootstrap replications. For phylogenies based on pathways, pairwise orthologs for each of the gene components of individual pathways were filtered out in all the *C. concisus* strains, and genes were then concatenated and aligned to the remaining strains using ClustalW [44].

For the SNP analysis, orthologs of individual gene components of selected KEGG pathways found conserved across all *C. concisus* genomes were aligned using local ClustalW alignment software. Non-synonymous SNP changes (resulting in amino acid changes) which displayed grouping by known phenotype categorisations were filtered out using customised python scripts. Pfam domain analysis of the proteins containing these SNPs was performed using Interproscan [45] integrated in Blast2GO.

### Availability of supporting data

Draft genomes of the *Campylobacter concisus* strains sequenced in this study have been deposited in GenBank available at the National Center for Biotechnology Information (http://www.ncbi.nlm.nih.gov/), and accession numbers of these genomes are provided in Table 1. All other supporting data are included as additional files.

## Competing interests

The authors declare that they have no competing interests.

## Authors’ contributions

MRW and HMM conceived the idea; NOK cultured the bacteria and extracted the DNA; NPD assembled and annotated the genomes; NPD and MRW performed the bioinformatic analyses; NOK and HMM performed the biological analyses; NOK, NPD, MRW and HMM drafted the manuscript. All authors read and approved the final manuscript.

## Supplementary Material

Additional file 1: Table S1 Syntenical placement of the UNSWCD plasmid genes across the *C. concisus* strains. A group of eight UNSWCD plasmid genes including the exotoxin 9 displayed syntenical conservation and are highlighted in grey. **Table S2.** KEGG pathways which show the same sub-set of genes conserved across all eight *C. concisus* strains. **Table S3.** Genes found specific to individual *Campylobacter concisus* strains are tabulated below and those found in syntenic blocks are highlighted in grey. **Table S4.** Genes found specific to *C. concisus* strain-pairs are tabulated below and those placed syntenically are highlighted in grey. **Table S5.** Percentage of genes specific to each *Campylobacter concisus* strain. **Table S6.** Pathway-specific differences within sequenced *C. concisus* strains and across *C. concisus* and *C. jejuni* species. **Figure S1.** Phylogenetic analyses of the different pathways within the eight *C. concisus* strains. a) Bile efflux; b) flagellar biosynthesis; c) chemotaxis; d) lipopolysaccharide biosynthesis; e) Sulfur relay system; and f) N-glycosylation. Click here for file
