# Supplementary material for: Comparative genomics of Campylobacter concisus isolates reveals genetic diversity and provides insights into disease association
Source: BMC Genomics. 2013 Aug 28;14:585. doi: 10.1186/1471-2164-14-585 (PMC3765806; doi:10.1186/1471-2164-14-585)
Supplement: Additional file 1: Table S1 — Syntenical placement of the UNSWCD plasmid genes across the C. concisus strains. A group of eight UNSWCD plasmid genes including the exotoxin 9 displayed syntenical conservation and are highlighted in grey. Table S2. KEGG pathways which show the same sub-set of genes conserved across all eight C. concisus strains. Table S3. Genes found specific to individual Campylobacter concisus strains are tabulated below and those found in syntenic blocks are highlighted in grey. Table S4. Genes found specific to C. concisus strain-pairs are tabulated below and those placed syntenically are highlighted in grey. Table S5. Percentage of genes specific to each Campylobacter concisus strain. Table S6. Pathway-specific differences within sequenced C. concisus strains and across C. concisus and C. jejuni species. Figure S1. Phylogenetic analyses of the different pathways within the eight C. concisus strains. a) Bile efflux; b) flagellar biosynthesis; c) chemotaxis; d) lipopolysaccharide biosynthesis; e) Sulfur relay system; and f) N-glycosylation. [file 1471-2164-14-585-S1.docx]

**Table S1** Syntenical placement of the UNSWCD plasmid genes across the *C. concisus* strains. A group of eight UNSWCD plasmid genes including the exotoxin 9 displayed syntenical conservation and are highlighted in grey.

1. **Plasmid from UNSWCD**

| **Gene ID/Description** | **Scaffold** | **Position co-ordinates** | **Orientation** |
| --- | --- | --- | --- |
| **UNSW2** |  |  |  |
| UNSWCD_11\|hypothetical protein | 8 | 66131, 65691 | + |
| UNSWCD_18\|StbE | 8 | 69276, 69028 | + |
| UNSWCD_25\|StbE replicon stabilization toxin | 8 | 69553, 69269 | + |
| UNSWCD_8\|MobA-like protein | 8 | 71891, 70302 | + |
| UNSWCD_14\|exotoxin 9 | 8 | 73405, 73097 | - |
| UNSWCD_23\|R.Ecl18kI | 8 | 76956, 76321 | - |
| UNSWCD_7\|DNA-cytosine methyltransferase | 8 | 78237, 77059 | + |
| UNSWCD_13\|site-specific recombinase2C resolvase family2C | 8 | 78807, 78502 | - |
| UNSWCD_19\|Site-specific recombinase | 8 | 80085, 79132 | - |
| **UNSW3** |  |  |  |
| UNSWCD_10\|hypothetical protein | 10 | 45655, 45533 | + |
| UNSWCD_11\|hypothetical protein | 10 | 50533, 50012 | - |
| UNSWCD_9\|Mature parasite-infected erythrocyte surface antigen | 10 | 52411, 51002 | - |
| UNSWCD_1\|hypothetical protein | 10 | 53390, 52686 | - |
| UNSWCD_3\|hypothetical protein | 10 | 56622, 55723 | - |
| UNSWCD_22\|Sodium/solute symporter | 10 | 58007, 56625 | - |
| UNSWCD_15\|hypothetical protein | 10 | 58429, 58010 | - |
| UNSWCD_20\|Radical SAM | 10 | 59382, 58450 | - |
| UNSWCD_4\|Fe-S oxidoreductase | 10 | 60341, 59382 | - |
| UNSWCD_16\|glycosyl transferase2C family 2 | 10 | 61023, 60322 | - |
| UNSWCD_12\|Membrane spanning protein | 10 | 61654, 60995 | - |
| UNSWCD_26\|DedA family inner membrane protein YdjZ | 10 | 62342, 61635 | - |
| UNSWCD_6\|Choline kinase | 10 | 63850, 62342 | - |
| UNSWCD_21\|hypothetical protein | 10 | 64224, 63850 | - |
| UNSWCD_24\|hypothetical protein | 10 | 64862, 64227 | - |
| UNSWCD_17\|hypothetical protein | 10 | 67299, 65332 | + |
| UNSWCD_18\|StbE | 10 | 67632, 67384 | + |
| UNSWCD_25\|StbE replicon stabilization toxin | 10 | 67909, 67625 | + |
| UNSWCD_8\|MobA-like protein | 10 | 70224, 68635 | + |
| UNSWCD_14\|exotoxin 9 | 10 | 71738, 71430 | - |
| UNSWCD_23\|R.Ecl18kI | 10 | 75318, 74404 | - |
| UNSWCD_7\|DNA-cytosine methyltransferase | 10 | 76599, 75421 | + |
| UNSWCD_13\|site-specific recombinase2C resolvase family2C | 10 | 77174, 76869 | - |
| UNSWCD_19\|Site-specific recombinase | 10 | 78440, 77499 | - |
| **UNSW1** |  |  |  |
| UNSWCD_9\|Mature parasite-infected erythrocyte surface antigen | 13 | 3143, 1734 | - |
| UNSWCD_1\|hypothetical protein | 13 | 4131, 3427 | - |
| UNSWCD_15\|hypothetical protein | 13 | 8607, 8188 | - |
| UNSWCD_20\|Radical SAM | 13 | 9559, 8627 | - |
| UNSWCD_4\|Fe-S oxidoreductase | 13 | 10521, 9559 | - |
| UNSWCD_2\|hypothetical protein | 13 | 11178, 10567 | - |
| UNSWCD_16\|glycosyl transferase2C family 2 | 13 | 11904, 11203 | - |
| UNSWCD_12\|Membrane spanning protein | 13 | 12535, 11876 | - |
| UNSWCD_26\|DedA family inner membrane protein YdjZ | 13 | 13223, 12516 | - |
| UNSWCD_6\|Choline kinase | 13 | 14731, 13223 | - |
| UNSWCD_21\|hypothetical protein | 13 | 15105, 14731 | - |
| UNSWCD_24\|hypothetical protein | 13 | 15743, 15108 | - |
| UNSWCD_17\|hypothetical protein | 13 | 18173, 16215 | + |
| UNSWCD_18\|StbE | 13 | 18507, 18259 | + |
| UNSWCD_25\|StbE replicon stabilization toxin | 13 | 18784, 18500 | + |
| UNSWCD_8\|MobA-like protein | 13 | 21120, 19528 | + |
| UNSWCD_14\|exotoxin 9 | 13 | 22639, 22331 | - |
| UNSWCD_23\|R.Ecl18kI | 13 | 26484, 25579 | - |
| UNSWCD_7\|DNA-cytosine methyltransferase | 13 | 27791, 26613 | + |
| UNSWCD_13\|site-specific recombinase2C resolvase family2C | 13 | 28361, 28056 | - |
| UNSWCD_19\|Site-specific recombinase | 13 | 29640, 28660 | - |
| UNSWCD_11\|hypothetical protein | 3 | 68423, 67749 | + |
| **UNSWCS** |  |  |  |
| UNSWCD_7\|DNA-cytosine methyltransferase | 1 | 49030, 48335 | - |
| UNSWCD_11\|hypothetical protein | 10 | 64873, 64433 | + |
| UNSWCD_18\|StbE | 10 | 68007, 67759 | + |
| UNSWCD_25\|StbE replicon stabilization toxin | 10 | 68284, 68000 | + |
| UNSWCD_9\|Mature parasite-infected erythrocyte surface antigen | 10 | 69682, 68885 | + |
| **ATCC 51562** |  |  |  |
| UNSWCD_11\|hypothetical protein | 3 | 261351, 260929 | + |
| UNSWCD_9\|Mature parasite-infected erythrocyte surface antigen | 3 | 266894, 265332 | - |
| UNSWCD_25\|StbE replicon stabilization toxin | 3 | 267495, 267211 | - |
| UNSWCD_18\|StbE | 3 | 267736, 267488 | - |
| UNSWCD_7\|DNA-cytosine methyltransferase | 3 | 276317, 275316 | - |
| UNSWCD_17\|hypothetical protein | 3 | 277450, 276551 | - |
| UNSWCD_5\|TonB-dependent receptor | 3 | 306958, 305537 | + |
| **ATCC 51561** |  |  |  |
| UNSWCD_9\|Mature parasite-infected erythrocyte surface antigen | 10 | 4607, 3204 | - |
| UNSWCD_1\|hypothetical protein | 10 | 5596, 4892 | - |
| UNSWCD_3\|hypothetical protein | 10 | 6551, 5634 | - |
| UNSWCD_22\|Sodium/solute symporter | 10 | 7954, 6551 | - |
| UNSWCD_15\|hypothetical protein | 10 | 8376, 7957 | - |
| UNSWCD_20\|Radical SAM | 10 | 9328, 8396 | - |
| UNSWCD_4\|Fe-S oxidoreductase | 10 | 10287, 9328 | - |
| UNSWCD_16\|glycosyl transferase2C family 2 | 10 | 10969, 10268 | - |
| UNSWCD_12\|Membrane spanning protein | 10 | 11600, 10941 | - |
| UNSWCD_8\|MobA-like protein | 13 | 12577, 11612 | + |
| UNSWCD_26\|DedA family inner membrane protein YdjZ | 10 | 12288, 11581 | - |
| UNSWCD_6\|Choline kinase | 10 | 13796, 12288 | - |
| UNSWCD_21\|hypothetical protein | 10 | 14170, 13796 | - |
| UNSWCD_24\|hypothetical protein | 10 | 14808, 14173 | - |
| UNSWCD_11\|hypothetical protein | 7 | 126329, 125916 | + |
| UNSWCD_17\|hypothetical protein | 7 | 173836, 171911 | + |
| **BAA-1457** |  |  |  |
| UNSWCD_18\|StbE | NC_009802.1 | 343056, 342835 | + |
| UNSWCD_17\|hypothetical protein | NC_009802.1 | 346650, 344533 | + |

1. **Plasmids from BAA-1457**

| **pCCON31** | | | | |
| --- | --- | --- | --- | --- |
| **Gene ID** | **Description** | **Contig** | **Position co-ordinates** | **Orientation** |
| **UNSW2** |  |  |  |  |
| YP_001456653.1 | hypothetical protein | 40 | 549, 1 | + |
| YP_001456658.1 | hypothetical protein | 103 | 193, 2 | + |
| YP_001456659.1 | hypothetical protein | 64 | 544, 218 | - |
| YP_001456657.1 | putative phytanoyl-CoAdioxygenase | 102 | 1014, 709 | - |
| YP_001456654.1 | hypothetical protein | 30 | 14386, 13778 | + |
| YP_001456648.1 | hypothetical protein | 1 | 49107, 48466 | - |
| YP_001456650.1 | putative periplasmic protein | 5 | 332965, 331196 |  |
| YP_001456651.1 | filamentous haemagglutinin family protein | 5 | 336571, 332978 | + |
|  |  |  |  |  |
| **UNSW3** |  |  |  |  |
| YP_001456654.1 | hypothetical protein | 13 | 19909, 19301 | + |
|  |  |  |  |  |
| **UNSW1** |  |  |  |  |
| YP_001456650.1 | putative periplasmic protein | 20 | 2310, 541 | + |
| YP_001456651.1 | filamentous haemagglutinin family protein | 20 | 5742, 2323 | + |
| YP_001456654.1 | hypothetical protein | 1 | 9056, 8448 | - |
| YP_001456661.1 | hypothetical protein | 13 | 22688, 20388 | + |
|  |  |  |  |  |
| **UNSWCS** |  |  |  |  |
| YP_001456650.1 | putative periplasmic protein | 86 | 1014, 1 | + |
| YP_001456658.1 | hypothetical protein | 96 | 190, 2 | - |
| YP_001456659.1 | hypothetical protein | 181 | 255, 1 | - |
| YP_001456649.1 | hypothetical protein | 15 | 552, 334 | - |
| YP_001456661.1 | hypothetical protein | 21 | 3120, 817 | + |
| YP_001456648.1 | hypothetical protein | 15 | 1241, 558 | - |
| YP_001456657.1 | putative phytanoyl-CoAdioxygenase | 67 | 1530, 1159 | - |
| YP_001456656.1 | hypothetical protein | 67 | 2029, 1589 | - |
| YP_001456655.1 | hypothetical protein | 67 | 2169, 2032 | - |
| YP_001456651.1 | filamentous haemagglutinin family protein | 75 | 2288, 828 | - |
| YP_001456647.1 | Cpp22 | 15 | 2877, 1588 | - |
| YP_001456653.1 | hypothetical protein | 67 | 3755, 2931 | - |
| YP_001456662.1 | hypothetical protein | 21 | 6179, 5964 | - |
| YP_001456663.1 | hypothetical protein | 21 | 6649, 6197 | - |
| YP_001456654.1 | hypothetical protein | 90 | 9306, 8698 | - |
| YP_001456665.1 | hypothetical protein | 21 | 23344, 23204 | - |
| YP_001456666.1 | HipA domain-containing protein | 21 | 34089, 32920 | - |
| YP_001456669.1 | hypothetical protein | 21 | 35238, 35131 | - |
| YP_001456672.1 | phage integrase family site-specific recombinase | 21 | 36120, 35137 | - |
| YP_001456673.1 | hypothetical protein | 21 | 36510, 36409 | + |
| YP_001456674.1 | hypothetical protein | 21 | 38190, 37936 | + |
| YP_001456675.1 | hypothetical protein | 21 | 39557, 38361 | + |
| YP_001456678.1 | hypothetical protein3 | 21 | 41064, 40213 | + |
| YP_001456679.1 | hypothetical protein | 21 | 41462, 41067 | + |
|  |  |  |  |  |
| **ATCC51562** |  |  |  |  |
| YP_001456648.1 | hypothetical protein | 10 | 40472, 39831 | - |
| YP_001456661.1 | hypothetical protein | 9 | 183476, 180759 | + |
|  |  |  |  |  |
| **ATCC51561** |  |  |  |  |
| YP_001456661.1 | hypothetical protein | 45 | 876, 10 | + |
| YP_001456650.1 | putative periplasmic protein | 9 | 3939, 2170 | + |
| YP_001456651.1 | filamentous haemagglutinin family protein | 9 | 7380, 3952 | + |
| YP_001456652.1 | hypothetical protein | 9 | 14216, 13905 | + |
|  |  |  |  |  |
| **pCCON16** | | | | |
|  |  |  |  |  |
| **UNSW2** |  |  |  |  |
| YP_001456694.1 | hypothetical protein | 43 | 769, 17 | + |
| YP_001456682.1 | hypothetical protein | 29 | 1176, 37 | + |
| YP_001456686.1 | hypothetical protein | 113 | 1279, 161 | + |
| YP_001456690.1 | hypothetical protein | 23 | 44106, 43078 | + |
| YP_001456701.1 | hypothetical protein | 6 | 70248, 69952 | + |
| YP_001456702.1 | mobilization protein | 6 | 71288, 70302 | + |
|  |  |  |  |  |
| **UNSW3** |  |  |  |  |
| YP_001456686.1 | hypothetical protein | 43 | 782, 3 | + |
| YP_001456694.1 | hypothetical protein | 50 | 1571, 783 | + |
| YP_001456690.1 | hypothetical protein | 47 | 903, 286 | - |
| YP_001456682.1 | hypothetical protein | 36 | 1133, 3 | - |
| YP_001456693.1 | hypothetical protein | 31 | 11476, 11099 | + |
| YP_001456701.1 | hypothetical protein | 20 | 14132, 13836 | + |
| YP_001456702.1 | mobilization protein | 20 | 15127, 14186 | + |
|  |  |  |  |  |
| **UNSW1** |  |  |  |  |
| YP_001456682.1 | hypothetical protein | 65 | 1407, 268 | + |
| YP_001456694.1 | hypothetical protein | 49 | 779, 3 | - |
| YP_001456686.1 | hypothetical protein | 47 | 1020, 1 | - |
| YP_001456690.1 | hypothetical protein | 11 | 1679, 651 | - |
| YP_001456701.1 | hypothetical protein | 18 | 19474, 19178 | + |
| YP_001456702.1 | mobilization protein | 18 | 20514, 19528 | + |
|  |  |  |  |  |
| **UNSWCS** |  |  |  |  |
| YP_001456686.1 | hypothetical protein | 190 | 894, 1 | + |
| YP_001456682.1 | hypothetical protein | 192 | 403, 2 | + |
| YP_001456694.1 | hypothetical protein | 122 | 961, 266 | - |
| YP_001456690.1 | hypothetical protein | 4 | 61886, 60858 | + |
|  |  |  |  |  |
| **ATCC51562** |  |  |  |  |
| YP_001456690.1 | hypothetical protein | 1 | 81348, 80782 | - |
| YP_001456682.1 | hypothetical protein | 1 | 81519, 80809 | - |
| YP_001456686.1 | hypothetical protein | 3 | 162724, 162038 | - |
|  |  |  |  |  |
| **ATCC51561** |  |  |  |  |
| YP_001456680.1 | putative pNL871104_protein | 130 | 1120, 164 | + |
| YP_001456691.1 | bacteriocin-type signal sequence domain-containing protein | 30 | 1619, 1194 | + |
| YP_001456690.1 | hypothetical protein | 30 | 2719, 1691 | + |
| YP_001456702.1 | mobilization protein 22 | 22 | 2051, 192 | - |
| YP_001456701.1 | hypothetical protein | 22 | 2358, 2044 | - |
| YP_001456700.1 | hypothetical protein | 22 | 2527, 2348 | - |
| YP_001456699.1 | hypothetical protein | 22 | 2992, 2738 | + |
| YP_001456683.1 | Cpp12 | 30 | 3529, 2969 | + |
| YP_001456681.1 | hypothetical protein | 22 | 3532, 3128 | + |
| YP_001456684.1 | hypothetical protein | 30 | 3798, 3532 | + |
| YP_001456685.1 | hypothetical protein | 30 | 4401, 3844 | + |
| YP_001456686.1 | hypothetical protein | 30 | 5641, 4460 | + |
| YP_001456696.1 | HmcD domain-containing protein | 22 | 5736, 4885 | + |
| YP_001456695.1 | peptidase family protein | 22 | 6314, 5724 | + |
| YP_001456693.1 | hypothetical protein | 30 | 6405, 5908 | + |
| YP_001456694.1 | hypothetical protein | 27 | 9961, 9182 | - |
| YP_001456682.1 | hypothetical protein | 12 | 125517, 124378 | - |

**Table S2** KEGG pathways which show the same sub-set of genes conserved across all eight *C. concisus* strains.

| **KEGG ID** | **Pathway name** | **Number of pathway gene components conserved across all *C. concisus* strains** |
| --- | --- | --- |
| 00780 | Biotin metabolism | 7 |
| 00550 | Peptidoglycan biosynthesis | 15 |
| 00730 | Thiamine metabolism | 11 |
| 00970 | Aminoacyl-tRNA biosynthesis | 47 |
| 00430 | Taurine and hypotaurine metabolism | 3 |
| 00562 | Inositol phosphate metabolism | 3 |
| 00860 | Porphyrin and chlorophyll metabolism | 11 |
| 00410 | beta-Alanine metabolism | 3 |
| 02040 | Flagellar assembly | 28 |
| 00471 | D-Glutamine and D-glutamate metabolism | 5 |
| 00472 | D-Arginine and D-ornithine metabolism | 2 |
| 00473 | D-Alanine metabolism | 4 |
| 00290 | Valine, leucine and isoleucine biosynthesis | 11 |
| 03420 | Nucleotide excision repair | 9 |
| 03440 | Homologous recombination | 18 |
| 03030 | DNA replication | 14 |
| 00920 | Sulfur metabolism | 11 |
| 00625 | Chloroalkane and chloroalkene degradation | 3 |
| 00071 | Fatty acid metabolism | 3 |
| 00627 | Aminobenzoate degradation | 3 |
| 00740 | Riboflavin metabolism | 7 |
| 00660 | C5-Branched dibasic acid metabolism | 7 |
| 00760 | Nicotinate and nicotinamide metabolism | 10 |
| 00310 | Lysine degradation | 4 |
| 00450 | Selenocompound metabolism | 9 |
| 00020 | Citrate cycle (TCA cycle) | 23 |
| 00130 | Ubiquinone and other terpenoid-quinone biosynthesis | 6 |
| 00052 | Galactose metabolism | 4 |
| 00230 | Purine metabolism | 46 |
| 00750 | Vitamin B6 metabolism | 6 |
| 00790 | Folate biosynthesis | 16 |
| 03060 | Protein export | 17 |
| 03410 | Base excision repair | 11 |
| 01040 | Biosynthesis of unsaturated fatty acids | 3 |
| 00401 | Novobiocin biosynthesis | 4 |
| 00400 | Phenylalanine, tyrosine and tryptophan biosynthesis | 18 |
| 00540 | Lipopolysaccharide biosynthesis | 22 |
| 00360 | Phenylalanine metabolism | 7 |
| 00362 | Benzoate degradation | 4 |
| 03020 | RNA polymerase | 5 |
| 00280 | Valine, leucine and isoleucine degradation | 2 |
| 04122 | Sulfur relay system | 10 |
| 00650 | Butanoate metabolism | 11 |
| 02030 | Bacterial chemotaxis | 13 |
| 00770 | Pantothenate and CoA biosynthesis | 15 |
| 00061 | Fatty acid biosynthesis | 12 |
| 00523 | Polyketide sugar unit biosynthesis | 3 |
| 00030 | Pentose phosphate pathway | 12 |
| 00460 | Cyanoamino acid metabolism | 4 |
| 00300 | Lysine biosynthesis | 14 |
| 00500 | Starch and sucrose metabolism | 6 |
| 00260 | Glycine, serine and threonine metabolism | 18 |
| 00380 | Tryptophan metabolism | 3 |

**Table S3** Genes found specific to individual *Campylobacter concisus* strains are tabulated below and those found in syntenic blocks are highlighted in grey.

| ***Campylobacter concisus* UNSWCD** | |
| --- | --- |
| fig\|6666666.462.peg.202 | atp-dependent protease atp-binding subunit |
| fig\|6666666.462.peg.252 | hypothetical protein |
| fig\|6666666.462.peg.336 | lipopolysaccharide biosynthesis protein |
| fig\|6666666.462.peg.341 | glycosyl transferase group 1 |
| fig\|6666666.462.peg.342 | glycosyl group 1 family protein |
| fig\|6666666.462.peg.343 | o-antigen polymerase |
| fig\|6666666.462.peg.344 | glycosyltransferase |
| fig\|6666666.462.peg.345 | heparinase ii iii-like protein |
| fig\|6666666.462.peg.346 | polysaccharide biosynthesis protein |
| fig\|6666666.462.peg.347 | phosphatase |
| fig\|6666666.462.peg.348 | aminotransferase |
| fig\|6666666.462.peg.349 | capk protein |
| fig\|6666666.462.peg.382 | methyl-accepting chemotaxis protein |
| fig\|6666666.462.peg.405 | hypothetical protein |
| fig\|6666666.462.peg.654 | hi0933 family partial |
| fig\|6666666.462.peg.659 | membrane protein |
| fig\|6666666.462.peg.660 | tir protein |
| fig\|6666666.462.peg.745 | surface protein |
| fig\|6666666.462.peg.1170 | integrase family protein |
| fig\|6666666.462.peg.1216 | hypothetical protein |
| fig\|6666666.462.peg.1360 | hypothetical protein |
| fig\|6666666.462.peg.1490 | dna mismatch repair protein |
| fig\|6666666.462.peg.1491 | dna-cytosine methyltransferase |
| ***Campylobacter concisus* UNSW2** | |
| fig\|6666666.10684.peg.40 | licd2 protein |
| fig\|6666666.10684.peg.41 | Hypothetical protein |
| fig\|6666666.10684.peg.42 | integral membrane protein domain protein |
| fig\|6666666.10684.peg.43 | transcriptional choline ethanolamine kinase |
| fig\|6666666.10684.peg.600 | dgqhr domain protein |
| fig\|6666666.10684.peg.601 | adenine-specific dna-methyltransferase |
| fig\|6666666.10684.peg.788 | hypothetical protein |
| fig\|6666666.10684.peg.789 | restriction endonuclease family protein |
| fig\|6666666.10684.peg.902 | 1 aminotransferase |
| fig\|6666666.10684.peg.903 | hypothetical protein |
| fig\|6666666.10684.peg.904 | haloacid dehalogenase-like hydrolase |
| fig\|6666666.10684.peg.905 | cyclase family protein |
| fig\|6666666.10684.peg.906 | nad-dependent epimerase dehydratase |
| fig\|6666666.10684.peg.908 | acetyltransferase (isoleucine patch superfamily) |
| fig\|6666666.10684.peg.909 | udp-glucose 4-epimerase |
| fig\|6666666.10684.peg.910 | transferase hexapeptide repeat containing protein |
| fig\|6666666.10684.peg.911 | 3-deoxy-manno-octulosonate cytidylyltransferase |
| fig\|6666666.10684.peg.912 | 2-dehydro-3-deoxyglucarate aldolase |
| fig\|6666666.10684.peg.1075 | arginase |
| fig\|6666666.10684.peg.1084 | hypothetical protein |
| fig\|6666666.10684.peg.1559 | hypothetical protein |
| fig\|6666666.10684.peg.1561 | tetratricopeptide repeat domain protein |
| fig\|6666666.10684.peg.1840 | atpase family protein |
| fig\|6666666.10684.peg.1875 | cation diffusion facilitator family transporter |
| fig\|6666666.10684.peg.1892 | hnh endonuclease |
| fig\|6666666.10684.peg.2014 | 3-oxoacyl-(acyl carrier protein) synthase iii |
| fig\|6666666.10684.peg.2015 | gnat family |
| fig\|6666666.10684.peg.2017 | 3-oxoacyl-acp reductase |
| fig\|6666666.10684.peg.2018 | short-chain dehydrogenase reductase sdr |
| fig\|6666666.10684.peg.2020 | amino acid adenylation domain protein |
| fig\|6666666.10684.peg.2021 | methyltransferase family |
| fig\|6666666.10684.peg.2022 | amp-dependent synthetase and ligase |
| fig\|6666666.10684.peg.2023 | nad dependent epimerase dehydratase family protein |
| fig\|6666666.10684.peg.2025 | metallo-beta-lactamase domain protein |
| fig\|6666666.10684.peg.2026 | cytidylyltransferase domain protein |
| ***Campylobacter concisus* UNSW3** | |
| fig\|6666666.10685.peg.231 | rna methylase |
| fig\|6666666.10685.peg.232 | hypothetical protein |
| fig\|6666666.10685.peg.656 | replication initiation protein |
| fig\|6666666.10685.peg.1004 | phage-related protein |
| fig\|6666666.10685.peg.1006 | phage-related protein |
| fig\|6666666.10685.peg.1166 | glycosyl transferase family 2 |
| fig\|6666666.10685.peg.1167 | probable k5 antigen synthesis |
| fig\|6666666.10685.peg.1168 | conserved domain protein |
| fig\|6666666.10685.peg.1169 | lipopolysaccharide biosynthesis protein |
| fig\|6666666.10685.peg.1171 | formyl transferase |
| fig\|6666666.10685.peg.1173 | fucosyl transferase |
| fig\|6666666.10685.peg.1174 | methyltransferase family |
| fig\|6666666.10685.peg.1175 | polysaccharide biosynthesis protein |
| fig\|6666666.10685.peg.1176 | 2-aminoethylphosphonate aminotransferase |
| fig\|6666666.10685.peg.1177 | phosphonopyruvate decarboxylase |
| fig\|6666666.10685.peg.1179 | phosphoenolpyruvate phosphomutase |
| fig\|6666666.10685.peg.1246 | atp-dependent endonuclease |
| fig\|6666666.10685.peg.1293 | hypothetical protein |
| fig\|6666666.10685.peg.1911 | hypothetical protein |
| fig\|6666666.10685.peg.1912 | hypothetical protein |
| fig\|6666666.10685.peg.1923 | site-specific phage integrase family |
| fig\|6666666.10685.peg.1924 | protein |
| fig\|6666666.10685.peg.1925 | membrane protein |
| fig\|6666666.10685.peg.1926 | phage protein |
| fig\|6666666.10685.peg.1927 | hypothetical protein |
| fig\|6666666.10685.peg.1948 | SigF |
| fig\|6666666.10685.peg.1949 | uncharacterized conserved protein |
| ***Campylobacter concisus* UNSW1** | |
| fig\|6666666.18222.peg.367 | f0f1-type atp synthase |
| fig\|6666666.18222.peg.368 | hypothetical protein |
| fig\|6666666.18222.peg.427 | thiosulfate sulfur transferase |
| fig\|6666666.18222.peg.428 | hypothetical protein |
| fig\|6666666.18222.peg.531 | dna methylase n-4 n-6 domain protein |
| fig\|6666666.18222.peg.532 | ulcer-associated gene restriction endonuclease |
| fig\|6666666.18222.peg.720 | aldo keto reductase |
| fig\|6666666.18222.peg.845 | didehydrogluconate reductase |
| fig\|6666666.18222.peg.911 | type iii site-specific deoxyribonuclease |
| fig\|6666666.18222.peg.1208 | cog1691: ncair mutase -related proteins |
| fig\|6666666.18222.peg.1305 | gnat family |
| fig\|6666666.18222.peg.1306 | hypothetical protein |
| fig\|6666666.18222.peg.1311 | transposase is204 is1001 is1096 is1165 protein |
| fig\|6666666.18222.peg.1408 | transcriptional activator |
| fig\|6666666.18222.peg.1473 | hypothetical protein |
| fig\|6666666.18222.peg.1744 | outer membrane adhesin-like protein |
| fig\|6666666.18222.peg.1799 | methyltransferase type 12 |
| fig\|6666666.18222.peg.1811 | hypothetical protein |
| fig\|6666666.18222.peg.1813 | glycosyl group 1 family protein |
| fig\|6666666.18222.peg.1814 | acyltransferase family protein |
| fig\|6666666.18222.peg.1815 | polysaccharide pyruvyl transferase |
| fig\|6666666.18222.peg.1816 | capsule polysaccharide biosynthesis protein |
| fig\|6666666.18222.peg.1817 | integral membrane protein |
| fig\|6666666.18222.peg.1820 | membrane- metal-dependent hydrolase |
| fig\|6666666.18222.peg.1821 | glycosyl transferase family 2 |
| fig\|6666666.18222.peg.1899 | hypothetical protein |
|  | |
| ***Campylobacter concisus* UNSWCS** | |
| fig\|6666666.10687.peg.106 | hypothetical protein |
| fig\|6666666.10687.peg.286 | abi-like protein |
| fig\|6666666.10687.peg.301 | dna methylase |
| fig\|6666666.10687.peg.571 | restriction endonuclease |
| fig\|6666666.10687.peg.572 | resolvase domain containing protein |
| fig\|6666666.10687.peg.574 | transcriptional regulator |
| fig\|6666666.10687.peg.576 | crispr-associated protein motif protein |
| fig\|6666666.10687.peg.577 | hypothetical protein |
| fig\|6666666.10687.peg.578 | crispr-associated protein |
| fig\|6666666.10687.peg.580 | crispr-associated ramp protein |
| fig\|6666666.10687.peg.581 | conserved domain protein |
| fig\|6666666.10687.peg.582 | crispr-associated ramp protein |
| fig\|6666666.10687.peg.583 | crispr-associated tm1811 family |
| fig\|6666666.10687.peg.584 | hypothetical protein |
| fig\|6666666.10687.peg.586 | crispr-associated protein cas1 |
| fig\|6666666.10687.peg.587 | crispr-associated protein cas6 |
| fig\|6666666.10687.peg.589 | gtp-binding protein |
| fig\|6666666.10687.peg.590 | small gtp-binding protein domain protein |
| fig\|6666666.10687.peg.592 | hypothetical protein |
| fig\|6666666.10687.peg.594 | gtp-binding protein |
| fig\|6666666.10687.peg.596 | atp gtp binding protein |
| fig\|6666666.10687.peg.597 | cell division protein |
| fig\|6666666.10687.peg.598 | transcriptional regulator |
| fig\|6666666.10687.peg.603 | filamentation induced by camp protein fic |
| fig\|6666666.10687.peg.604 | hypothetical protein |
| fig\|6666666.10687.peg.610 | phage repressor |
| fig\|6666666.10687.peg.669 | hypothetical protein |
| fig\|6666666.10687.peg.700 | pf03235 family protein |
| fig\|6666666.10687.peg.705 | hypothetical protein |
| fig\|6666666.10687.peg.761 | transcriptional |
| fig\|6666666.10687.peg.763 | hypothetical protein |
| fig\|6666666.10687.peg.908 | hypothetical protein |
| fig\|6666666.10687.peg.909 | adenylylsulfate kinase |
| fig\|6666666.10687.peg.912 | molybdenum cofactor biosynthesis protein |
| fig\|6666666.10687.peg.979 | mafb alternative c-terminus |
| fig\|6666666.10687.peg.1104 | cytochrome c-type heme-binding periplasmic protein |
| fig\|6666666.10687.peg.1247 | pf06114 domain protein |
| fig\|6666666.10687.peg.1365 | hypothetical protein |
| fig\|6666666.10687.peg.1398 | hypothetical protein |
| fig\|6666666.10687.peg.1484 | filamentous hemagglutinin family omp |
| fig\|6666666.10687.peg.1485 | filamentous hemagglutinin omp |
| fig\|6666666.10687.peg.1901 | adhesin hemagglutinin |
| fig\|6666666.10687.peg.1907 | appr-1-p processing domain protein |
| fig\|6666666.10687.peg.1908 | appr-1-p processing domain-containing protein |
| fig\|6666666.10687.peg.1914 | gp19 |
| fig\|6666666.10687.peg.1921 | phage terminase large subunit |
| fig\|6666666.10687.peg.1922 | phage structural protein |
| fig\|6666666.10687.peg.1923 | phage mu protein f-like protein |
| fig\|6666666.10687.peg.1924 | hypothetical protein |
| fig\|6666666.10687.peg.1925 | major capsid protein |
| fig\|6666666.10687.peg.1927 | structural phage protein |
| fig\|6666666.10687.peg.1935 | phage tape measure protein |
| fig\|6666666.10687.peg.1936 | tail length tape measure partial |
| fig\|6666666.10687.peg.1938 | phage minor tail protein l |
| fig\|6666666.10687.peg.1941 | tail fiber truncation |
| fig\|6666666.10687.peg.1984 | phage-associated protein |
| fig\|6666666.10687.peg.1985 | RloG protein, putative |
| fig\|6666666.10687.peg.2044 | d-arabino-heptulosonate 7-phosphate synthase |
| fig\|6666666.10687.peg.2057 | hypothetical protein |
| fig\|6666666.10687.peg.2120 | signal peptide protein |
| ***Campylobacter concisus* ATCC 51562** | |
| fig\|6666666.10688.peg.8 | nitrilase cyanide hydratase |
| fig\|6666666.10688.peg.11 | general stress protein 14 |
| fig\|6666666.10688.peg.62 | toxin-antitoxin antitoxin component |
| fig\|6666666.10688.peg.103 | hypothetical protein |
| fig\|6666666.10688.peg.153 | outer membrane autotransporter barrel protein |
| fig\|6666666.10688.peg.173 | abortive phage infection |
| fig\|6666666.10688.peg.174 | hypothetical protein |
| fig\|6666666.10688.peg.175 | endonuclease |
| fig\|6666666.10688.peg.176 | dna mismatch repair enzyme (atpase) |
| fig\|6666666.10688.peg.181 | mu c-terminal domain protein |
| fig\|6666666.10688.peg.182 | dna transposition protein |
| fig\|6666666.10688.peg.226 | 4-carboxymuconolactone decarboxylase |
| fig\|6666666.10688.peg.250 | methionyl-trna formyltransferase |
| fig\|6666666.10688.peg.255 | nad-binding domain protein |
| fig\|6666666.10688.peg.257 | acetyltransferase |
| fig\|6666666.10688.peg.258 | flagellin modification |
| fig\|6666666.10688.peg.260 | conserved domain protein |
| fig\|6666666.10688.peg.263 | methyltransferase type 11 |
| fig\|6666666.10688.peg.264 | glycosyl transferase |
| fig\|6666666.10688.peg.397 | aminobenzoyl-glutamate transport protein |
| fig\|6666666.10688.peg.420 | phenylalanine and histidine ammonia-lyase |
| fig\|6666666.10688.peg.421 | outer membrane domain protein |
| fig\|6666666.10688.peg.513 | nucleotide-binding protein |
| fig\|6666666.10688.peg.718 | hypothetical protein |
| fig\|6666666.10688.peg.818 | protein containing duf88 |
| fig\|6666666.10688.peg.834 | kap family p-loop domain protein |
| fig\|6666666.10688.peg.937 | family protein |
| fig\|6666666.10688.peg.1047 | type i restriction-modification system |
| fig\|6666666.10688.peg.1108 | thiazole biosynthesis protein |
| fig\|6666666.10688.peg.1109 | dependent receptor |
| fig\|6666666.10688.peg.1110 | periplasmic binding protein |
| fig\|6666666.10688.peg.1113 | methyltransferase domain protein |
| fig\|6666666.10688.peg.1114 | hypothetical protein |
| fig\|6666666.10688.peg.1115 | hypothetical protein |
| fig\|6666666.10688.peg.1125 | tail collar domain protein |
| fig\|6666666.10688.peg.1130 | hypothetical protein |
| fig\|6666666.10688.peg.1132 | hit family protein |
| fig\|6666666.10688.peg.1133 | bacteriophage head to tail connecting protein |
| fig\|6666666.10688.peg.1135 | terminase b |
| fig\|6666666.10688.peg.1137 | yqey family protein |
| fig\|6666666.10688.peg.1140 | d12 adenine-specific dna methyltransferase |
| fig\|6666666.10688.peg.1142 | bacteriophage protein |
| fig\|6666666.10688.peg.1151 | hypothetical protein |
| fig\|6666666.10688.peg.1153 | sel1 repeat protein |
| fig\|6666666.10688.peg.1163 | hypothetical protein |
| fig\|6666666.10688.peg.1165 | phage protein |
| fig\|6666666.10688.peg.1207 | phosphate binding protein |
| fig\|6666666.10688.peg.1208 | phosphate abc permease protein |
| fig\|6666666.10688.peg.1209 | phosphate abc permease protein |
| fig\|6666666.10688.peg.1224 | putative lipoprotein |
| fig\|6666666.10688.peg.1232 | outer membrane autotransporter barrel protein |
| fig\|6666666.10688.peg.1365 | pf03235 family protein |
| fig\|6666666.10688.peg.1463 | heme iron utilization protein |
| fig\|6666666.10688.peg.1466 | hemin uptake system hemin-binding protein |
| fig\|6666666.10688.peg.1467 | oxygen-independent coproporphyrinogen iii oxidase |
| fig\|6666666.10688.peg.1468 | flavodoxin family protein |
| fig\|6666666.10688.peg.1481 | c4-dicarboxylate malic acid transporter |
| fig\|6666666.10688.peg.1482 | transcriptional crp fnr family protein |
| fig\|6666666.10688.peg.1483 | sulfide:quinone reductase |
| fig\|6666666.10688.peg.1608 | hnh endonuclease |
| fig\|6666666.10688.peg.1695 | rhs repeat-associated core domain protein |
| fig\|6666666.10688.peg.1811 | integral membrane protein |
| fig\|6666666.10688.peg.1813 | hypothetical exported 24-amino acid repeat protein |
| fig\|6666666.10688.peg.1873 | hypothetical protein |
| ***Campylobacter concisus* ATCC 51561** | |
| fig\|6666666.18223.peg.376 | hypothetical protein |
| fig\|6666666.18223.peg.592 | helicase domain snf2 family domain protein |
| fig\|6666666.18223.peg.593 | pf14335 domain protein |
| fig\|6666666.18223.peg.595 | dna (cytosine-5-)-methyltransferase |
| fig\|6666666.18223.peg.618 | flavin reductase domain protein |
| fig\|6666666.18223.peg.811 | group 1 glycosyl transferase |
| fig\|6666666.18223.peg.866 | short-chain dehydrogenase reductase family |
| fig\|6666666.18223.peg.1107 | family 2 glycosyl transferase |
| fig\|6666666.18223.peg.1111 | conserved domain protein |
| fig\|6666666.18223.peg.1112 | tigr04325 family |
| fig\|6666666.18223.peg.1113 | glycosyltransferase |
| fig\|6666666.18223.peg.1116 | glucose-1-phosphate thymidylyltransferase |
| fig\|6666666.18223.peg.1117 | dtdp-glucose -dehydratase |
| fig\|6666666.18223.peg.1218 | hypothetical protein |
| fig\|6666666.18223.peg.1242 | hypothetical protein |
| fig\|6666666.18223.peg.1243 | muramidase (phage lysozyme) |
| fig\|6666666.18223.peg.1244 | ef hand domain protein |
| fig\|6666666.18223.peg.1245 | hypothetical protein |
| fig\|6666666.18223.peg.1719 | putative lysozyme |
| fig\|6666666.18223.peg.1720 | conserved domain protein |
| ***Campylobacter concisus* BAA-1457** | |
| fig\|6666666.13147.peg.74 | ATPase |
| fig\|6666666.13147.peg.75 | smc domain protein |
| fig\|6666666.13147.peg.160 | hypothetical protein |
| fig\|6666666.13147.peg.225 | large extracellular alpha-helical protein |
| fig\|6666666.13147.peg.349 | hypothetical protein |
| fig\|6666666.13147.peg.359 | dna-cytosine methyltransferase |
| fig\|6666666.13147.peg.360 | dna-cytosine methyltransferase |
| fig\|6666666.13147.peg.361 | two component transcriptional regulator |
| fig\|6666666.13147.peg.362 | 5-methylcytosine-specific restriction enzyme b |
| fig\|6666666.13147.peg.363 | llaji restriction endonuclease |
| fig\|6666666.13147.peg.368 | biopolymer transport protein |
| fig\|6666666.13147.peg.376 | hypothetical protein |
| fig\|6666666.13147.peg.377 | gtp-binding protein |
| fig\|6666666.13147.peg.414 | glycosyl transferase group 1 |
| fig\|6666666.13147.peg.415 | membrane protein |
| fig\|6666666.13147.peg.416 | glycosyl transferase group 1 |
| fig\|6666666.13147.peg.738 | phenazine biosynthesis protein family protein |
| fig\|6666666.13147.peg.942 | two-component regulator |
| fig\|6666666.13147.peg.949 | 3-oxoacyl-[acyl-carrier-protein] synthase 3 |
| fig\|6666666.13147.peg.951 | zonula occludens toxin |
| fig\|6666666.13147.peg.952 | general secretion pathway protein d |
| fig\|6666666.13147.peg.954 | phage integrase |
| fig\|6666666.13147.peg.990 | diaminopimelate dehydrogenase |
| fig\|6666666.13147.peg.1540 | group 1 glycosyl transferase |
| fig\|6666666.13147.peg.1542 | group 1 glycosyl transferase |
| fig\|6666666.13147.peg.1543 | lipopolysaccharide n-acetylglucosaminyltransferase |
| fig\|6666666.13147.peg.1545 | group 1 glycosyl transferase |
| fig\|6666666.13147.peg.1546 | nad-dependent epimerase dehydratase |
| fig\|6666666.13147.peg.1547 | polysaccharide biosynthesis protein |
| fig\|6666666.13147.peg.1548 | group 1 glycosyl transferase |
| fig\|6666666.13147.peg.1549 | udp-glucuronic acid udp-4-keto-hexauronic acid |
| fig\|6666666.13147.peg.1550 | hypothetical protein |
| fig\|6666666.13147.peg.1551 | polysaccharide deacetylase |
| fig\|6666666.13147.peg.1552 | carbamoyl phosphate synthase-like-protein |
| fig\|6666666.13147.peg.1553 | mj0936 family |
| fig\|6666666.13147.peg.1554 | d-mycarose 3-c-methyltransferase |
| fig\|6666666.13147.peg.1557 | hypothetical protein |
| fig\|6666666.13147.peg.1558 | nad dependent epimerase dehydratase family protein |
| fig\|6666666.13147.peg.1560 | transferase hexapeptide repeat containing protein |
| fig\|6666666.13147.peg.1561 | oxidoreductase domain protein |
| fig\|6666666.13147.peg.1615 | general secretion pathway protein d |
| fig\|6666666.13147.peg.1616 | zonula occludens toxin |
| fig\|6666666.13147.peg.1618 | hypothetical protein |
| fig\|6666666.13147.peg.1624 | two-component regulator |
| fig\|6666666.13147.peg.1656 | abc permease protein |
| fig\|6666666.13147.peg.1657 | twin-arginine translocation pathway signal |
| fig\|6666666.13147.peg.1658 | carbon-monoxide catalytic subunit |
| fig\|6666666.13147.peg.1674 | transposase is116 is110 is902 family protein |
| fig\|6666666.13147.peg.1677 | hypothetical protein |
| fig\|6666666.13147.peg.1679 | dna-directed rna polymerase alpha chain |
| fig\|6666666.13147.peg.1680 | hypothetical protein |
| fig\|6666666.13147.peg.1681 | hypothetical protein |
| fig\|6666666.13147.peg.1683 | hypothetical protein |
| fig\|6666666.13147.peg.1769 | arylsulfatase |
| fig\|6666666.13147.peg.1829 | oxidoreductase family protein |
| fig\|6666666.13147.peg.1986 | chaperone protein |
| fig\|6666666.13147.peg.1987 | foldase protein |

**Table S4** Genes found specific to *C. concisus* strain-pairs are tabulated below and those placed syntenically are highlighted in grey.

| **Gene ID** | **Gene definition** | **Top Blast hit in NCBI nr database** |
| --- | --- | --- |
| **BAA-1457 & ATCC 51561** |  |  |
| fig\|6666666.13147.peg.272 | hypothetical protein | *Campylobacter concisus* BAA-1457 |
| fig\|6666666.13147.peg.327 | pyrimidine dimer dna glycosylase | *Campylobacter concisus* BAA-1457 |
| fig\|6666666.13147.peg.345 | aaa atpase | *Campylobacter concisus* BAA-1457 |
| fig\|6666666.13147.peg.346 | phage-related protein | *Campylobacter concisus* BAA-1457 |
| fig\|6666666.13147.peg.347 | transcriptional xre family | *Campylobacter concisus* BAA-1457 |
| fig\|6666666.13147.peg.364 | uncharacterized conserved protein | *Campylobacter concisus* BAA-1457 |
| fig\|6666666.13147.peg.375 | 3-isopropylmalate dehydrogenase | *Campylobacter concisus* BAA-1457 |
| fig\|6666666.13147.peg.860 | creatininase | *Campylobacter concisus* BAA-1457 |
| fig\|6666666.13147.peg.861 | cytosine permease | *Campylobacter concisus* BAA-1457 |
| fig\|6666666.13147.peg.1080 | peptidase family protein | *Campylobacter concisus* BAA-1457 |
| fig\|6666666.13147.peg.2002 | hypothetical protein | *Campylobacter concisus* BAA-1457 |
| **BAA-1457 & UNSW1** |  |  |
| fig\|6666666.13147.peg.163 | bifunctional dna-directed rna beta chain | *Campylobacter concisus* BAA-1457 |
| fig\|6666666.13147.peg.165 | hypothetical protein | *Campylobacter concisus* BAA-1457 |
| fig\|6666666.13147.peg.384 | trna 2-selenouridine synthase | *Campylobacter concisus* BAA-1457 |
| fig\|6666666.13147.peg.429 | transcriptional regulator | *Campylobacter concisus* BAA-1457 |
| fig\|6666666.13147.peg.431 | citrate transporter | *Campylobacter concisus* BAA-1457 |
| fig\|6666666.13147.peg.814 | addiction module txe family | *Campylobacter concisus* BAA-1457 |
| fig\|6666666.13147.peg.815 | addiction module family | *Campylobacter concisus* BAA-1457 |
| fig\|6666666.13147.peg.1133 | crispr-associated pf09484 family | *Campylobacter concisus* BAA-1457 |
| fig\|6666666.13147.peg.1135 | crispr-associated protein hmari subtype | *Campylobacter concisus* BAA-1457 |
| fig\|6666666.13147.peg.1555 | asparagine synthetase | *Campylobacter concisus* BAA-1457 |
| **UNSW2 & UNSW1** |  |  |
| fig\|6666666.10684.peg.137 | methyl-accepting chemotaxis protein | *Campylobacter* sp. 10_1_50 |
| fig\|6666666.10684.peg.1948 | membrane protein | *Campylobacter concisus* BAA-1457 |
| **UNSW2 & ATCC 51561** |  |  |
| fig\|6666666.10684.peg.699 | phosphoserine phosphatase | *Campylobacter* *showae* RM3277 |
| **UNSW2 & ATCC 51562** |  |  |
| fig\|6666666.10684.peg.630 | replication protein | *Campylobacter* *fetus* subsp. *venerealis* NCTC 10354 |
| fig\|6666666.10684.peg.632 | hypothetical protein | *Campylobacter* rectus RM3267 |
| fig\|6666666.10684.peg.635 | plasmid partitioning protein | *Campylobacter* rectus RM3267 |
| fig\|6666666.10684.peg.636 | modification methylase | *Campylobacter* rectus RM3267 |
| fig\|6666666.10684.peg.639 | hypothetical protein | *Campylobacter* *fetus* subsp. *venerealis* NCTC 10354 |
| fig\|6666666.10684.peg.640 | hypothetical protein | *Campylobacter* *rectus* RM3267 |
| fig\|6666666.10684.peg.641 | hypothetical protein | *Campylobacter* *fetus* subsp. *venerealis* NCTC 10354 |
| fig\|6666666.10684.peg.642 | hypothetical protein | *Campylobacter* *rectus* RM3267 |
| fig\|6666666.10684.peg.643 | 4 coupling protein | *Campylobacter* *rectus* RM3267 |
| fig\|6666666.10684.peg.644 | hypothetical protein | *Campylobacter* *rectus* RM3267 |
| fig\|6666666.10684.peg.645 | conjugative transfer regulon protein | *Campylobacter* *fetus* subsp. *venerealis* NCTC 10354 |
| fig\|6666666.10684.peg.647 | protein | *Campylobacter* *fetus* subsp. *venerealis* NCTC 10354 |
| fig\|6666666.10684.peg.648 | dna transformation compentancy | *Campylobacter* *rectus* RM3267 |
| fig\|6666666.10684.peg.649 | comb8 competence protein | *Campylobacter* *fetus* subsp. *venerealis* NCTC 10354 |
| fig\|6666666.10684.peg.650 | hypothetical protein | *Campylobacter* *fetus* subsp. *venerealis* NCTC 10354 |
| fig\|6666666.10684.peg.651 | dna transfer protein | *Campylobacter* *rectus* RM3267 |
| fig\|6666666.10684.peg.653 | relaxase mobilization nuclease domain-containing protein | *Campylobacter* *rectus* RM3267 |
| fig\|6666666.10684.peg.656 | hypothetical protein | *Campylobacter* *rectus* RM3267 |
| fig\|6666666.10684.peg.841 | bacteriophage-related integrase | *Campylobacter* *rectus* RM3267 |
| fig\|6666666.10684.peg.842 | cjp29-like protein | *Campylobacter* *rectus* RM3267 |
| fig\|6666666.10684.peg.843 | hypothetical protein | *Campylobacter* *rectus* RM3267 |
| fig\|6666666.10684.peg.844 | hypothetical protein | *Campylobacter* *fetus* subsp. *venerealis* NCTC 10354 |
| **UNSW2 & UNSWCS** |  |  |
| fig\|6666666.10684.peg.365 | ggdef family protein | *Shewanella sediminis* HAW-EB3 |
| fig\|6666666.10684.peg.440 | tetratricopeptide repeat domain protein | *Neisseria wadsworthii* 9715 |
| fig\|6666666.10684.peg.584 | hypothetical protein | *Campylobacter* *gracilis* RM3268 |
| fig\|6666666.10684.peg.588 | hypothetical protein | *Campylobacter* *gracilis* RM3268 |
| fig\|6666666.10684.peg.589 | hypothetical protein | *Helicobacter bilis* ATCC 43879 |
| fig\|6666666.10684.peg.662 | protein of unknown function DUF262 | *Clostridium carboxidivorans* P7 |
| fig\|6666666.10684.peg.663 | protein of hypothetical function duf262 | *Arcobacter butzleri* ED-1 |
| fig\|6666666.10684.peg.684 | hypothetical protein | *Pseudoalteromonas sp*. BSi20495 |
| fig\|6666666.10684.peg.686 | appr-1-p processing domain protein | *Nautilia profundicola* AmH |
| fig\|6666666.10684.peg.702 | phage protein | *Hydrogenobacter thermophilus* TK6 |
| fig\|6666666.10684.peg.705 | bacteriophage head-tail connecting protein | *Campylobacter* *coli* 2680 |
| fig\|6666666.10684.peg.708 | major head protein | *Hydrogenobacter thermophilus* TK6 |
| fig\|6666666.10684.peg.710 | tail fiber protein | *Campylobacter* sp. FOBRC14 |
| fig\|6666666.10684.peg.715 | hypothetical protein | *Nitratifractor salsuginis* DSM 16511 |
| fig\|6666666.10684.peg.716 | hypothetical protein | *Campylobacter* sp. FOBRC14 |
| fig\|6666666.10684.peg.717 | lipoprotein | *Campylobacter* *rectus* RM3267 |
| fig\|6666666.10684.peg.718 | hypothetical protein | *Campylobacter* sp. FOBRC14 |
| fig\|6666666.10684.peg.721 | hypothetical protein | *Campylobacter* sp. FOBRC14 |
| fig\|6666666.10684.peg.723 | hypothetical protein | *Campylobacter* sp. FOBRC14 |
| fig\|6666666.10684.peg.730 | hypothetical protein | *Corynebacterium efficiens* YS-314 |
| fig\|6666666.10684.peg.792 | helix-turn-helix domain-containing protein | *Geobacter sp*. M21 |
| fig\|6666666.10684.peg.797 | phage plasmid p4 family | *Sulfuricurvum kujiense* DSM 16994 |
| fig\|6666666.10684.peg.801 | hypothetical protein | *Fusobacterium sp.* 11_3_2 |
| fig\|6666666.10684.peg.845 | hypothetical protein | *Campylobacter* *gracilis* RM3268 |
| fig\|6666666.10684.peg.849 | tetratricopeptide tpr_2 repeat protein | *Trichoplax adhaerens* |
| fig\|6666666.10684.peg.896 | glycosyl family 2 | *Raphidiopsis brookii* D9 |
| fig\|6666666.10684.peg.899 | dtdp-4-dehydrorhamnose epimerase | *Sulfuricurvum kujiense* DSM 16994 |
| fig\|6666666.10684.peg.900 | cdp-glucose -dehydratase | *Thiovulum sp*. ES |
| fig\|6666666.10684.peg.901 | glucose-1-phosphate cytidylyltransferase | *Sulfuricurvum kujiense* DSM 16994 |
| fig\|6666666.10684.peg.913 | polysaccharide biosynthesis protein | *Sulfuricurvum kujiense* DSM 16994 |
| fig\|6666666.10684.peg.914 | tigr04331 family | *Thiovulum sp*. ES |
| fig\|6666666.10684.peg.916 | acylneuraminate cytidylyltransferase | *Desulfovibrio vulgaris* str. Hildenborough |
| fig\|6666666.10684.peg.917 | cytidylyltransferase domain protein | *Thiovulum sp*. ES |
| fig\|6666666.10684.peg.919 | iron-containing alcohol dehydrogenase | *Thiovulum sp*. ES |
| fig\|6666666.10684.peg.1015 | dynamin family protein | *Pseudomonas sp*. PAMC 25886 |
| fig\|6666666.10684.peg.1016 | elongation factor tu gtp binding protein | *Helicobacter pylori* F57 |
| fig\|6666666.10684.peg.1314 | hypothetical protein | *Campylobacter concisus* BAA-1457 |
| fig\|6666666.10684.peg.1649 | restriction endonuclease | *Campylobacter* *coli* LMG 23336 |
| fig\|6666666.10684.peg.1650 | type ii modification enzyme | *Campylobacter* *coli* LMG 23336 |
| **ATCC 51562 & ATCC 51561** |  |  |
| fig\|6666666.10688.peg.265 | group 2 family protein | *Methylomicrobium alcaliphilum* 20Z |
| fig\|6666666.10688.peg.339 | toxin-antitoxin antitoxin xre family | *Campylobacter* *gracilis* RM3268 |
| fig\|6666666.10688.peg.941 | abc transporter | *Helicobacter pullorum* MIT 98-5489 |
| fig\|6666666.10688.peg.942 | hypothetical protein | *Campylobacter* *gracilis* RM3268 |
| fig\|6666666.10688.peg.1696 | hypothetical protein | *Campylobacter* *rectus* RM3267 |
| **UNSW2 & BAA-1457** |  |  |
| fig\|6666666.10684.peg.256 | putative lipoprotein | *Campylobacter concisus* BAA-1457 |
| fig\|6666666.10684.peg.676 | rare-cutting hnh restriction endonuclease paci | *Campylobacter* *showae* RM3277 |
| fig\|6666666.10684.peg.703 | recombination protein | *Campylobacter concisus* BAA-1457 |
| fig\|6666666.10684.peg.704 | hypothetical protein | *Campylobacter concisus* BAA-1457 |
| fig\|6666666.10684.peg.1049 | hypothetical protein | *Campylobacter concisus* BAA-1457 |
| fig\|6666666.10684.peg.1144 | pf10947 family protein | *Campylobacter concisus* BAA-1457 |
| fig\|6666666.10684.peg.1145 | hypothetical protein | *Campylobacter concisus* BAA-1457 |
| fig\|6666666.10684.peg.1146 | pf10947 family protein | *Campylobacter concisus* BAA-1457 |
| **ATCC 51612 & UNSW1** |  |  |
| fig\|6666666.10688.peg.914 | abc atp-binding protein | *Campylobacter* *curvus* 525.92 |
| fig\|6666666.10688.peg.917 | phage colicin resistance and tellurite resistance protein | *Campylobacter* *showae* RM3277 |
| fig\|6666666.10688.peg.1814 | anaerobic c4-dicarboxylate uptake c family protein | *Campylobacter* *rectus* RM3267 |
| fig\|6666666.10688.peg.1815 | oligoendopeptidase f | *Campylobacter* *gracilis* RM3268 |
| **UNSWCD & UNSW3** |  |  |
| fig\|6666666.462.peg.748 | conserved domain protein | *Campylobacter concisus* UNSWCD |
| fig\|6666666.462.peg.930 | hypothetical protein | *Campylobacter concisus* UNSWCD |
| fig\|6666666.462.peg.940 | signal peptidase i | *Campylobacter concisus* UNSWCD |
| fig\|6666666.462.peg.1203 | phage-related protein | *Campylobacter concisus* UNSWCD |
| fig\|6666666.462.peg.1204 | hypothetical protein | *Campylobacter concisus* UNSWCD |
| fig\|6666666.462.peg.1205 | hypothetical protein | *Campylobacter concisus* UNSWCD |
| fig\|6666666.462.peg.1206 | uncharacterised protein | *Campylobacter concisus* UNSWCD |
| fig\|6666666.462.peg.1552 | hypothetical protein | *Campylobacter concisus* UNSWCD |
| fig\|6666666.462.peg.1553 | hypothetical protein | *Campylobacter concisus* UNSWCD |
| fig\|6666666.462.peg.1661 | hypothetical protein | *Campylobacter concisus* UNSWCD |
| **UNSWCD & UNSW1** |  |  |
| fig\|6666666.462.peg.1163 | hypothetical protein | *Peptostreptococcus anaerobius* 653-L |
| **UNSWCD & UNSWCS** |  |  |
| fig\|6666666.462.peg.992 | hypothetical protein | *Campylobacter concisus* UNSWCD |
| **UNSWCD & ATCC 51562** |  |  |
| fig\|6666666.462.peg.1154 | s-layer-rtx protein | *Campylobacter* sp. FOBRC14 |
| **UNSWCD & BAA-1457** |  |  |
| fig\|6666666.462.peg.784 | hypothetical protein | *Campylobacter concisus* UNSWCD |
| **ATCC 51562 & BAA-1457** |  |  |
| fig\|6666666.10688.peg.1320 | methyltransferase type 12 | *Campylobacter concisus* BAA-1457 |
| fig\|6666666.10688.peg.1321 | dependent receptor | *Campylobacter* sp. FOBRC14 |
| fig\|6666666.10688.peg.1480 | hypothetical protein | *Campylobacter concisus* BAA-1457 |
| **UNSW3 & UNSW1** |  |  |
| fig\|6666666.10685.peg.567 | mip family channel protein | *Finegoldia magna* ATCC 53516 |
| fig\|6666666.10685.peg.1417 | hypothetical protein | *Flavobacterium sp.* CF136 |
| fig\|6666666.10685.peg.1606 | arsenite methyltransferase | Finegoldia magna ATCC 29328 |
| fig\|6666666.10685.peg.1964 | protein | *Campylobacter* *rectus* RM3267 |
| **UNSW3 & UNSWCS** |  |  |
| fig\|6666666.10685.peg.1372 | hypothetical protein | *Campylobacter* sp. FOBRC14 |
| fig\|6666666.10685.peg.1373 | bacteriophage dna transposition protein | *Campylobacter* sp. FOBRC14 |
| fig\|6666666.10685.peg.1374 | bacteriophage dna transposition protein | *Campylobacter* sp. FOBRC14 |
| fig\|6666666.10685.peg.1381 | aaa domain protein | *Alteromonas macleodii* str. 'English Channel 673' |
| fig\|6666666.10685.peg.1382 | conserved domain protein | *Campylobacter* sp. FOBRC14 |
| fig\|6666666.10685.peg.1383 | mor transcription activator domain protein | *Campylobacter* sp. FOBRC14 |
| fig\|6666666.10685.peg.1384 | hypothetical protein | *Campylobacter* jejuni subsp. jejuni 414 |
| fig\|6666666.10685.peg.1385 | phage virion morphogenesis | *Campylobacter* sp. FOBRC14 |
| fig\|6666666.10685.peg.1386 | prophage f | *Campylobacter* sp. FOBRC14 |
| fig\|6666666.10685.peg.1387 | pf06074 family protein | *Campylobacter* sp. FOBRC14 |
| fig\|6666666.10685.peg.1388 | hypothetical protein | *Campylobacter* sp. FOBRC14 |
| fig\|6666666.10685.peg.1389 | atpase gpp-like protein | *Campylobacter* sp. FOBRC14 |
| fig\|6666666.10685.peg.1390 | conserved domain protein | *Campylobacter* sp. FOBRC14 |
| fig\|6666666.10685.peg.1391 | phage major capsid protein e | *Campylobacter* sp. FOBRC14 |
| fig\|6666666.10685.peg.1392 | hypothetical protein | *Campylobacter* sp. FOBRC14 |
| fig\|6666666.10685.peg.1393 | mu-like prophage i | *Campylobacter* sp. FOBRC14 |
| fig\|6666666.10685.peg.1394 | hypothetical protein | *Campylobacter* sp. FOBRC14 |
| fig\|6666666.10685.peg.1410 | mu-like prophage u protein gp41 | *Campylobacter* *gracilis* RM3268 |
| fig\|6666666.10685.peg.1936 | rare-cutting hnh restriction endonuclease paci | *Campylobacter* sp. 10_1_50 |
| **UNSW3 & ATCC 51562** |  |  |
| fig\|6666666.10685.peg.1404 | bacteriophage tail fiber protein | *Campylobacter* sp. FOBRC14 |
| **UNSW3 & ATCC 51561** |  |  |
| fig\|6666666.10685.peg.53 | type iii restriction-modification system enzyme res | *Caldithrix abyssi* DSM 13497 |
| fig\|6666666.10685.peg.873 | glycosyl family 9 | *Campylobacter* *rectus* RM3267 |
| fig\|6666666.10685.peg.875 | o-antigen polymerase family | *Campylobacter* sp. 10_1_50 |
| fig\|6666666.10685.peg.876 | glycosyl family 9 | *Campylobacter* sp. 10_1_50 |
| fig\|6666666.10685.peg.1118 | hypothetical protein | *Helicobacter pullorum* MIT 98-5489 |
| fig\|6666666.10685.peg.1164 | glycosyl transferase group 1 domain-containing membrane protein | *Caminibacter mediatlanticus* TB-2 |
| **UNSWCS & ATCC 51561** |  |  |
| fig\|6666666.10687.peg.934 | conserved hypothethical protein | *Campylobacter* sp. 10_1_50 |
| fig\|6666666.10687.peg.1323 | membrane protein | *Campylobacter* sp. 10_1_50 |
| **UNSWCS & BAA-1457** |  |  |
| fig\|6666666.10687.peg.2 | hypothetical protein | *Campylobacter concisus* BAA-1457 |
| fig\|6666666.10687.peg.284 | cobaltochelatase subunit | *Campylobacter concisus* BAA-1457 |
| **UNSWCS & ATCC 51562** |  |  |
| fig\|6666666.10687.peg.1232 | tigr01671 family protein | *Campylobacter* *rectus* RM3267 |
| fig\|6666666.10687.peg.1234 | hypothetical protein | *Sulfurimonas gotlandica* GD1 |
| fig\|6666666.10687.peg.1242 | recombinase rect protein | *Campylobacter* *gracilis* RM3268 |
| fig\|6666666.10687.peg.1243 | phage anti-repressor protein | *Campylobacter* *rectus* RM3267 |
| fig\|6666666.10687.peg.1245 | helix-turn-helix protein | *Campylobacter* *jejuni* subsp. *doylei* 269.97 |
| fig\|6666666.10687.peg.1902 | exonuclease 5 - | *Campylobacter* *gracilis* RM3268 |
| fig\|6666666.10687.peg.1911 | peptidase s24-like protein | *Campylobacter* sp. FOBRC14 |
| fig\|6666666.10687.peg.1913 | winged helix-turn-helix dna-binding protein | *Burkholderia phymatum* STM815 |
| **UNSW3 & BAA-1457** |  |  |
| fig\|6666666.10685.peg.229 | helix-turn-helix protein | *Campylobacter* *jejuni* subsp. *jejuni* 1854 |
| fig\|6666666.10685.peg.335 | gamma-glutamyl phosphate reductase | *Campylobacter concisus* BAA-1457 |
| fig\|6666666.10685.peg.662 | type i restriction modification dna specificity domain protein | *Desulfotomaculum carboxydivorans* CO-1-SRB |
| fig\|6666666.10685.peg.1931 | hypothetical protein | *Campylobacter* sp. 10_1_50 |
| fig\|6666666.10685.peg.1933 | hypothetical protein | *Campylobacter concisus* BAA-1457 |
| **UNSW1 & ATCC 51561** |  |  |
| fig\|6666666.18222.peg.242 | cytochrome c-type protein | *Campylobacter* *jejuni* subsp*. jejuni* LMG 23216 |
| fig\|6666666.18222.peg.244 | two component transcriptional winged helix family | *Campylobacter* *curvus* 525.92 |
| fig\|6666666.18222.peg.422 | type i site-specific family | *Campylobacter* sp. 10_1_50 |
| fig\|6666666.18222.peg.1804 | acyl dehydratase | *Francisella novicida* GA99-3548 |
| fig\|6666666.18222.peg.1806 | family protein | *Thioalkalimicrobium aerophilum* AL3 |

**Table S5** Percentage of genes specific to each *Campylobacter concisus* strain.

| **Disease type** | **Chronic** | | | | **Acute** | | | **Healthy** |
| --- | --- | --- | --- | --- | --- | --- | --- | --- |
| **Strain** | UNSW2 | UNSW3 | UNSWCD | UNSW1 | UNSWCS | ATCC 51562 | BAA-1457 | ATCC 51561 |
| **Strain-specific genes (%)** | 1.9 | 1.4 | 1.2 | 1.4 | 2.8 | 3.4 | 2.8 | 0.99 |

**Table S6** Pathway-specific differences within sequenced *C. concisus* strains and across *C. concisus* and *C. jejuni* species.

| **00680\|Methane metabolism** | | | | | | | | | | | | | | | | | | | | | | | |
| --- | --- | --- | --- | --- | --- | --- | --- | --- | --- | --- | --- | --- | --- | --- | --- | --- | --- | --- | --- | --- | --- | --- | --- |
|  | **cjj** | | | **cji** | | | **cjn** | **cjm** | **cjd** | **cje** | **cjr** | **cjs** | **cju** |  | **UNSWCD** | **UNSW2** | **UNSW3** | **UNSW1** | **UNSWCS** | **ATCC 51562** | **BAA-1457** | **ATCC 51561** |  |
| K03781 | Y | | | Y | | | Y | Y | Y | Y | Y | Y | Y |  | * | * | * | * | * | * | * | * | katE, CAT, catB, srpA; catalase [EC:1.11.1.6] |
| K03313 | Y | | | Y | | | Y | Y | Y | Y | Y | Y | Y |  | Y | * | Y | * | Y | Y | Y | Y | nhaA; Na+:H+ antiporter, NhaA family |
| K03315 | * | | | * | | | * | * | * | * | * | * | * |  | Y | Y | Y | Y | Y | * | Y | * | nhaC; Na+:H+ antiporter, NhaC family |
|  |  | | |  | | |  |  |  |  |  |  |  |  |  |  |  |  |  |  |  |  |  |
| **02030\|Bacterial chemotaxis** | | | | | | | | | | | | | | | | | | | | | | | |
|  | **cjj** | | | **cji** | | | **cjn** | **cjm** | **cjd** | **cje** | **cjr** | **cjs** | **cju** |  | **UNSWCD** | **UNSW2** | **UNSW3** | **UNSW1** | **UNSWCS** | **ATCC 51562** | **BAA-1457** | **ATCC 51561** |  |
| K03776 | * | | | * | | | * | * | * | * | * | * | * |  | * | * | * | * | * | * | * | Y | aer; aerotaxis receptor |
|  |  | | |  | | |  |  |  |  |  |  |  |  |  |  |  |  |  |  |  |  |  |
| **00330\|Arginine and proline metabolism** | | | | | | | | | | | | | | | | | | | | | | | |
|  | **cjj** | | | **cji** | | | **cjn** | **cjm** | **cjd** | **cje** | **cjr** | **cjs** | **cju** |  | **UNSWCD** | **UNSW2** | **UNSW3** | **UNSW1** | **UNSWCS** | **ATCC 51562** | **BAA-1457** | **ATCC 51561** |  |
| K00930 | Y | | | Y | | | Y | Y | Y | Y | Y | Y | Y |  | * | * | * | * | * | * | * | Y | argB; acetylglutamate kinase [EC:2.7.2.8] |
| K00145 | Y | | | Y | | | Y | Y | Y | Y | Y | Y | Y |  | * | * | * | * | * | * | * | Y | argC; N-acetyl-gamma-glutamyl-phosphate reductase [EC:1.2.1.38] |
| K00818 | Y | | | * | | | * | * | Y | Y | Y | * | Y |  | * | * | * | * | * | * | * | Y | argD; acetylornithine aminotransferase [EC:2.6.1.11] |
|  |  | | |  | | |  |  |  |  |  |  |  |  |  |  |  |  |  |  |  |  |  |
|  | | | | | | | | | | | | | | | | | | | | | | | |
| **00630\|Glyoxylate and dicarboxylate metabolism** | | | | | | | | | | | | | | | | | | | | | | | |
|  | **cjj** | | | **cji** | | | **cjn** | **cjm** | **cjd** | **cje** | **cjr** | **cjs** | **cju** |  | **UNSWCD** | **UNSW2** | **UNSW3** | **UNSW1** | **UNSWCS** | **ATCC 51562** | **BAA-1457** | **ATCC 51561** |  |
| K03779 | * | | | * | | | * | * | * | * | * | * | * |  | Y | * | Y | Y | * | * | Y | Y | ttdA; L(+)-tartrate dehydratase alpha subunit [EC:4.2.1.32] |
| K03780 | * | | | * | | | * | * | * | * | * | * | * |  | Y | * | Y | Y | * | * | Y | Y | ttdB; L(+)-tartrate dehydratase beta subunit [EC:4.2.1.32] |
| K00865 | * | | | * | | | * | * | * | * | * | * | * |  | Y | Y | Y | Y | Y | Y | Y | Y | glxK; glycerate kinase [EC:2.7.1.31] |
|  |  | | |  | | |  |  |  |  |  |  |  |  |  |  |  |  |  |  |  |  |  |
| **03070\|Bacterial secretion system** | | | | | | | | | | | | | | | | | | | | | | | |
|  | **cjj** | | | **cji** | | | **cjn** | **cjm** | **cjd** | **cje** | **cjr** | **cjs** | **cju** |  | **UNSWCD** | **UNSW2** | **UNSW3** | **UNSW1** | **UNSWCS** | **ATCC 51562** | **BAA-1457** | **ATCC 51561** |  |
| K03197 | Y | | | * | | | * | * | * | * | * | Y | * |  | * | * | * | * | * | * | * | * | virB2, lvhB2; type IV secretion system protein VirB2 |
| K03204 | Y | | | Y | | | Y | * | * | * | * | Y | * |  | * | Y | * | * | * | Y | * | Y | virB9, lvhB9; type IV secretion system protein VirB9 |
| K03201 | Y | | | Y | | | Y | * | * | * | * | Y | * |  | * | * | * | * | * | * | * | * | virB6, lvhB6; type IV secretion system protein VirB6 |
| K03203 | Y | | | Y | | | Y | * | * | * | * | Y | * |  | * | Y | * | * | * | Y | * | Y | virB8, lvhB8; type IV secretion system protein VirB8 |
| K03195 | Y | | | Y | | | Y | * | * | * | * | Y | * |  | * | Y | * | * | * | Y | * | Y | virB10, lvhB10; type IV secretion system protein VirB10 |
| K03199 | Y | | | Y | | | Y | * | * | * | * | Y | * |  | * | Y | * | * | * | Y | * | Y | virB4, lvhB4; type IV secretion system protein VirB4 |
| K03196 | Y | | | Y | | | Y | * | * | * | * | Y | * |  | * | Y | * | * | * | Y | * | Y | virB11, lvhB11; type IV secretion system protein VirB11 |
| K03205 | Y | | | Y | | | Y | * | * | * | * | Y | * |  | * | Y | * | * | * | Y | * | Y | virD4, lvhD4; type IV secretion system protein VirD4 |
| K11028 | * | | | * | | | * | * | * | * | * | * | * |  | * | * | * | * | * | * | * | * | vacA; vacuolating cytotoxin |
| K11903 | * | | | * | | | * | * | * | * | * | * | * |  | * | * | * | Y | * | * | Y | Y | K11903, hcp; type VI secretion system secreted protein Hcp |
| K11906 | * | | | * | | | * | * | * | * | * | * | * |  | * | * | * | Y | * | * | Y | Y | vasD, lip; type VI secretion system protein VasD |
| K11891 | * | | | * | | | * | * | * | * | * | * | * |  | * | * | * | Y | * | * | Y | Y | impL, vasK, icmF; type VI secretion system protein ImpL |
| K11892 | * | | | * | | | * | * | * | * | * | * | * |  | * | * | * | Y | * | * | Y | Y | impK, ompA, vasF, dotU; type VI secretion system protein ImpK |
| K12340 | * | | | * | | | * | * | * | * | * | * | * |  | * | * | * | * | * | * | * | * | tolC; outer membrane channel protein TolC |
| K03194 | * | | | * | | | * | * | * | * | * | * | * |  | * | * | * | * | * | * | * | Y | virB1; type IV secretion system protein VirB1 |
| K03200 | * | | | * | | | * | * | * | * | * | * | * |  | * | * | * | * | * | * | * | * | virB5, lvhB5; type IV secretion system protein VirB5 |
|  |  | | |  | | |  |  |  |  |  |  |  |  |  |  |  |  |  |  |  |  |  |
| **02020\|Two-component system** | | | | | | | | | | | | | | | | | | | | | | | |
|  | **cjj** | | | | **cji** | | **cjn** | **cjm** | **cjd** | **cje** | **cjr** | **cjs** | **cju** |  | **UNSWCD** | **UNSW2** | **UNSW3** | **UNSW1** | **UNSWCS** | **ATCC 51562** | **BAA-1457** | **ATCC 51561** |  |
| K11688 | * | | | | * | | * | * | * | * | * | * | * |  | Y | Y | Y | Y | Y | Y | Y | Y | dctP; C4-dicarboxylate-binding protein DctP |
| K11689 | * | | | | * | | * | * | * | * | * | * | * |  | Y | Y | Y | Y | Y | Y | Y | Y | dctQ; C4-dicarboxylate transporter, DctQ subunit |
| K11690 | * | | | | * | | * | * | * | * | * | * | * |  | Y | Y | Y | Y | Y | Y | Y | Y | dctM; C4-dicarboxylate transporter, DctM subunit |
| K08357 | * | | | | * | | * | * | * | * | * | * | * |  | * | * | * | Y | * | Y | Y | Y | ttrA; tetrathionate reductase subunit A |
| K08358 | * | | | | * | | * | * | * | * | * | * | * |  | * | * | * | Y | * | Y | Y | Y | ttrB; tetrathionate reductase subunit B |
|  |  | | | |  | |  |  |  |  |  |  |  |  |  |  |  |  |  |  |  |  |  |
| **00920\|Sulfur metabolism** | | | | | | | | | | | | | | | | | | | | | | | |
|  | **cjj** | | | **cji** | | | **cjn** | **cjm** | **cjd** | **cje** | **cjr** | **cjs** | **cju** |  | **UNSWCD** | **UNSW2** | **UNSW3** | **UNSW1** | **UNSWCS** | **ATCC 51562** | **BAA-1457** | **ATCC 51561** |  |
| K00641 | Y | | | * | | | * | * | * | * | Y | * | * |  | Y | Y | Y | Y | Y | Y | Y | Y | metX; homoserine O-acetyltransferase [EC:2.3.1.31] |
| K00380 | * | | | * | | | * | * | * | * | * | * | * |  | Y | Y | Y | Y | Y | Y | Y | Y | cysJ; sulfite reductase (NADPH) flavoprotein alpha-component [EC:1.8.1.2] |
| K14155 | * | | | * | | | * | * | * | * | * | * | * |  | Y | Y | Y | Y | Y | Y | Y | Y | patB, malY; cystathione beta-lyase [EC:4.4.1.8] |
|  |  | | |  | | |  |  |  |  |  |  |  |  |  |  |  |  |  |  |  |  |  |
| **04146\|Peroxisome** | | | | | | | | | | | | | | | | | | | | | | | |
|  | **cjj** | | | **cji** | | | **cjn** | **cjm** | **cjd** | **cje** | **cjr** | **cjs** | **cju** |  | **UNSWCD** | **UNSW2** | **UNSW3** | **UNSW1** | **UNSWCS** | **ATCC 51562** | **BAA-1457** | **ATCC 51561** |  |
| K04565 | * | | | * | | | * | * | * | * | * | * | * |  | Y | Y | Y | Y | Y | Y | Y | Y | sodC, SOD1; Cu/Zn superoxide dismutase [EC:1.15.1.1] |
| K04564 | Y | | | Y | | | Y | Y | Y | Y | Y | Y | Y |  | Y | Y | Y | Y | Y | Y | Y | Y | sodA, sodB, SOD2; superoxide dismutase, Fe-Mn family [EC:1.15.1.1] |
|  |  | | |  | | |  |  |  |  |  |  |  |  |  |  |  |  |  |  |  |  |  |
| **00550\|Peptidoglycan biosynthesis** | | | | | | | | | | | | | | | | | | | | | | | |
|  | **cjj** | | | **cji** | | | **cjn** | **cjm** | **cjd** | **cje** | **cjr** | **cjs** | **cju** |  | **UNSWCD** | **UNSW2** | **UNSW3** | **UNSW1** | **UNSWCS** | **ATCC 51562** | **BAA-1457** | **ATCC 51561** |  |
| K05367 | * | | | * | | | * | * | * | * | * | * | * |  | Y | Y | Y | Y | Y | Y | Y | Y | pbpC; penicillin-binding protein 1C [EC:2.4.1.-] |
|  |  | | |  | | |  |  |  |  |  |  |  |  |  |  |  |  |  |  |  |  |  |
| **00250\|Alanine, aspartate and glutamate metabolism** | | | | | | | | | | | | | | | | | | | | | | | |
|  | | | **cjj** | | | **cji** | **cjn** | **cjm** | **cjd** | **cje** | **cjr** | **cjs** | **cju** |  | **UNSWCD** | **UNSW2** | **UNSW3** | **UNSW1** | **UNSWCS** | **ATCC 51562** | **BAA-1457** | **ATCC 51561** |  |
| K00266 | | | Y | | | Y | Y | Y | Y | Y | Y | Y | Y |  | * | * | * | * | * | * | * | * | gltD; glutamate synthase (NADPH/NADH) small chain [EC:1.4.1.13 1.4.1.14] |
| K13821 | | | Y | | | Y | Y | Y | Y | Y | Y | Y | Y |  | * | * | * | * | * | * | * | * | putA; proline dehydrogenase / delta 1-pyrroline-5-carboxylate dehydrogenase [EC:1.5.99.8 1.5.1.12] |
| K00262 | | | * | | | * | * | * | * | * | * | * | * |  | Y | Y | Y | Y | Y | Y | Y | Y | gdhA; glutamate dehydrogenase (NADP+) [EC:1.4.1.4] |
|  | | |  | | |  |  |  |  |  |  |  |  |  |  |  |  |  |  |  |  |  |  |
| **00910\|Nitrogen metabolism** | | | | | | | | | | | | | | | | | | | | | | | |
|  | | | **cjj** | | | **cji** | **cjn** | **cjm** | **cjd** | **cje** | **cjr** | **cjs** | **cju** |  | **UNSWCD** | **UNSW2** | **UNSW3** | **UNSW1** | **UNSWCS** | **ATCC 51562** | **BAA-1457** | **ATCC 51561** |  |
| K03385 | | | Y | | | Y | Y | Y | Y | Y | Y | Y | Y |  | * | * | * | * | * | * | * | Y | nrfA; cytochrome c-552 [EC:1.7.2.2] |
| K15876 | | | Y | | | Y | Y | Y | Y | Y | Y | Y | Y |  | * | * | * | * | * | * | * | Y | nrfH; cytochrome c-type protein |
| K01501 | | | Y | | | * | * | * | * | Y | Y | * | * |  | Y | Y | Y | Y | Y | Y | Y | Y | nitrilase [EC:3.5.5.1] |
| K05601 | | | * | | | * | * | * | * | * | * | * | * |  | Y | Y | Y | Y | Y | * | Y | Y | hcp; hydroxylamine reductase [EC:1.7.99.1] |
| K10535 | | | * | | | * | * | * | * | * | * | * | * |  | Y | Y | Y | Y | Y | Y | Y | Y | hao; hydroxylamine oxidase [EC:1.7.3.4] |
| K04561 | | | * | | | * | * | * | * | * | * | * | * |  | Y | Y | Y | Y | Y | Y | Y | Y | norB; nitric oxide reductase subunit B [EC:1.7.2.5] |
| K00376 | | | * | | | * | * | * | * | * | * | * | * |  | Y | Y | Y | Y | Y | Y | Y | Y | nosZ; nitrous-oxide reductase [EC:1.7.2.4] |
| K04014 | | | * | | | * | * | * | * | * | * | * | * |  | Y | Y | Y | Y | Y | Y | Y | Y | nrfC; protein NrfC |
| K04015 | | | * | | | * | * | * | * | * | * | * | * |  | Y | Y | Y | Y | Y | Y | Y | Y | nrfD; protein NrfD |
| K02569 | | | * | | | * | * | * | * | * | * | * | * |  | Y | Y | Y | Y | Y | Y | Y | Y | napC; cytochrome c-type protein NapC |
|  | | |  | | |  |  |  |  |  |  |  |  |  |  |  |  |  |  |  |  |  |  |
| **00860\|Porphyrin and chlorophyll metabolism** | | | | | | | | | | | | | | | | | | | | | | | |
|  | | **cjj** | | | | **cji** | **cjn** | **cjm** | **cjd** | **cje** | **cjr** | **cjs** | **cju** |  | **UNSWCD** | **UNSW2** | **UNSW3** | **UNSW1** | **UNSWCS** | **ATCC 51562** | **BAA-1457** | **ATCC 51561** |  |
| K02217 | | Y | | | | Y | Y | Y | Y | Y | Y | Y | Y |  | * | * | * | * | * | * | * | * | ftnA, ftn; ferritin [EC:1.16.3.1] |
|  | |  | | | |  |  |  |  |  |  |  |  |  |  |  |  |  |  |  |  |  |  |
| **02010\|ABC transporters** | | | | | | | | | | | | | | | | | | | | | | | |
|  | | **cjj** | | | | **cji** | **cjn** | **cjm** | **cjd** | **cje** | **cjr** | **cjs** | **cju** |  | **UNSWCD** | **UNSW2** | **UNSW3** | **UNSW1** | **UNSWCS** | **ATCC 51562** | **BAA-1457** | **ATCC 51561** |  |
| K02012 | | Y | | | | Y | Y | Y | Y | Y | Y | Y | Y |  | * | * | * | * | * | * | * | * | afuA, fbpA; iron(III) transport system substrate-binding protein |
| K02011 | | Y | | | | Y | Y | Y | Y | Y | Y | Y | Y |  | * | * | * | * | * | * | * | * | afuB, fbpB; iron(III) transport system permease protein |
| K02010 | | Y | | | | Y | Y | Y | Y | Y | Y | Y | Y |  | * | * | * | * | * | * | * | * | afuC, fbpC; iron(III) transport system ATP-binding protein [EC:3.6.3.30] |
| K02040 | | Y | | | | Y | Y | Y | Y | Y | Y | Y | Y |  | * | * | * | * | * | Y | * | Y | pstS; phosphate transport system substrate-binding protein |
| K02037 | | Y | | | | Y | Y | Y | Y | Y | Y | Y | Y |  | * | * | * | * | * | Y | * | Y | pstC; phosphate transport system permease protein |
| K02038 | | Y | | | | Y | Y | Y | Y | Y | Y | Y | Y |  | * | * | * | * | * | Y | * | Y | pstA; phosphate transport system permease protein |
| K02036 | | Y | | | | Y | * | Y | Y | Y | Y | Y | Y |  | * | * | * | * | * | Y | * | Y | pstB; phosphate transport system ATP-binding protein [EC:3.6.3.27] |
| K01999 | | Y | | | | Y | Y | Y | Y | Y | Y | Y | Y |  | * | * | * | * | * | * | * | * | livK; branched-chain amino acid transport system substrate-binding protein |
| K01997 | | Y | | | | Y | Y | Y | Y | Y | Y | Y | Y |  | * | * | * | * | * | * | * | * | livH; branched-chain amino acid transport system permease protein |
| K01998 | | Y | | | | Y | Y | Y | Y | Y | Y | Y | Y |  | * | * | * | * | * | * | * | * | livM; branched-chain amino acid transport system permease protein |
| K01995 | | Y | | | | Y | Y | Y | Y | Y | Y | Y | Y |  | * | * | * | * | * | * | * | * | livG; branched-chain amino acid transport system ATP-binding protein |
| K01996 | | Y | | | | Y | Y | Y | Y | Y | Y | Y | Y |  | * | * | * | * | * | * | * | * | livF; branched-chain amino acid transport system ATP-binding protein |
| K10107 | | Y | | | | Y | Y | Y | Y | Y | Y | Y | Y |  | * | * | * | * | * | * | * | * | ABC-2.CPSE.P1; capsular polysaccharide transport system permease protein |
| K09688 | | Y | | | | Y | Y | Y | Y | Y | Y | Y | Y |  | * | * | * | * | * | * | * | * | ABC-2.CPSE.P; capsular polysaccharide transport system permease protein |
| K09689 | | Y | | | | Y | Y | Y | Y | Y | Y | Y | Y |  | * | * | * | * | * | * | * | * | ABC-2.CPSE.A; capsular polysaccharide transport system ATP-binding protein |
| K09687 | | * | | | | * | * | * | * | * | * | * | * |  | Y | Y | Y | Y | Y | Y | Y | Y | ABC-2.AB.A; antibiotic transport system ATP-binding protein |

**Figure S1** Phylogenetic analyses of the different pathways within the eight *C. concisus* strains. a) Bile efflux; b) flagellar biosynthesis; c) chemotaxis; d) lipopolysaccharide biosynthesis; e) Sulfur relay system; and f) N-glycosylation.

| **(A)** 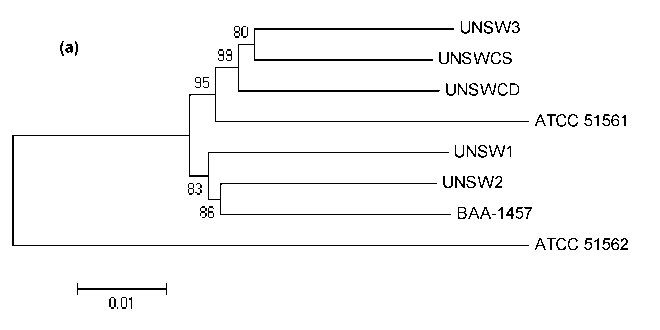 | **(B)** 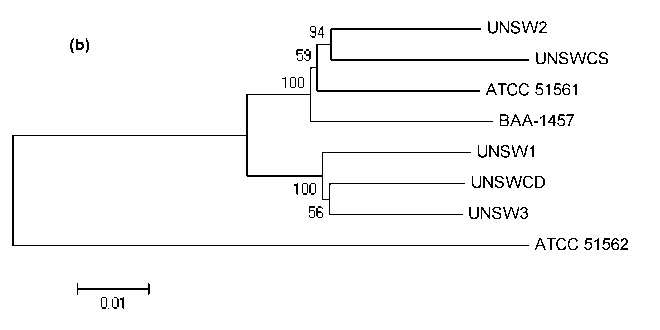 | **(C)** 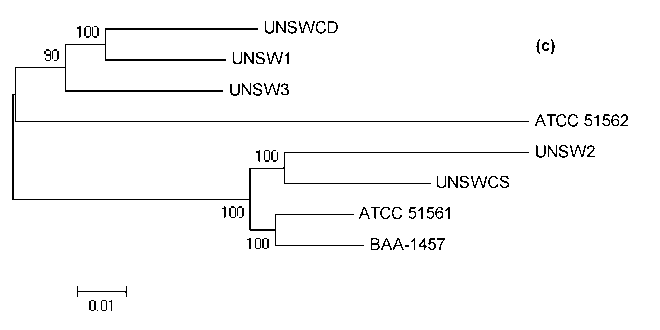 |
| --- | --- | --- |
| **(D)** 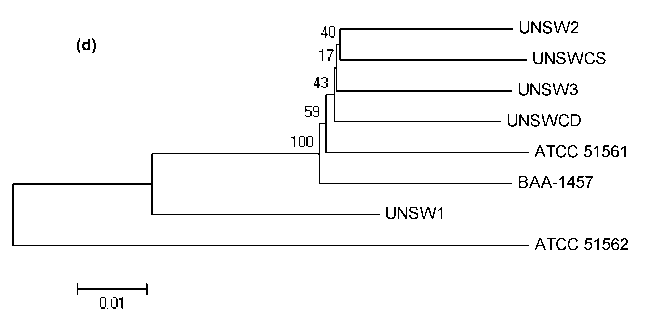 | **(E)** 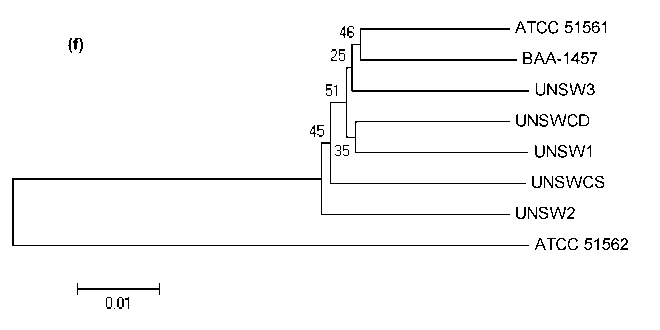 | **(F)** 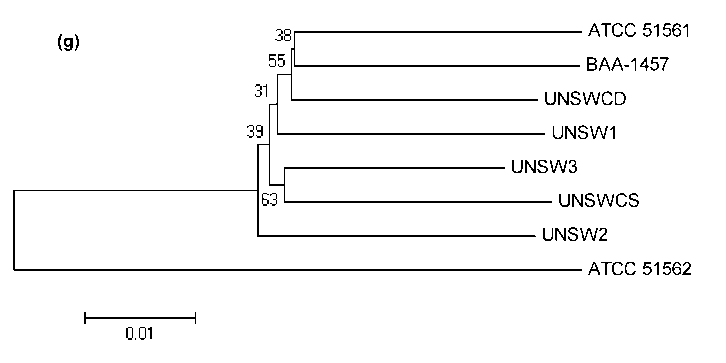 |
